# Perturbation of mitochondrial Ca^2+^ homeostasis activates cross-compartmental proteostatic response in Arabidopsis

**DOI:** 10.1007/s44154-026-00314-4

**Published:** 2026-05-28

**Authors:** Xiaoyan Zhang, Chongyang Ma, Xinyue Bao, Shenyu Zhang, Omar Zayed, Zhengjing Zhang, Kai Tang, Shaojun Xie, Yunsheng Wang, Dayong Zhang, Huawei Xu, Huifang Jia, Xinying Wang, Qianyan Lei, Xiaocui Wang, Junli Zhang, Savithramma P. Dinesh-Kumar, Chun-Peng Song, Jian-Kang Zhu, Xiaohong Zhu

**Affiliations:** 1 State Key Laboratory of Crop Stress Adaptation and Improvement, School of Life Sciences, Henan University, Kaifeng, 475004 China; 2 State Key Laboratory of Bio-Breeding and Integrated Utilization, Henan University, Kaifeng, 475004 China; 3 Department of Horticulture and Landscape Architecture, Purdue University, West Lafayette, IN 47907 USA; 4 Genetics Department, Faculty of Agriculture, Menoufia University, Menoufia, Egypt; 5 CAS Center for Excellence in Molecular Plant Sciences, Chinese Academy of Sciences, Shanghai, 201602 China; 6 Department of Plant Biology and The Genome Center, College of Biological Sciences, University of California, Davis, CA 95616 USA; 7 Institute of Advanced Biotechnology, Institute of Homeostatic Medicine, and School of Medicine, Southern University of Science and Technology, Shenzhen, 518055 China; 8 Key Laboratory of Gene Editing Technologies (Hainan), Ministry of Agricultural and Rural Affairs/National Nanfan Research Institute (Sanya), Chinese Academy of Agricultural Sciences, Sanya, 572024 China; 9 Faculty of Medicine, Macau University of Science and Technology, Taipa, Macau 999078 China

**Keywords:** Mitochondrial Ca^2+^ homeostasis, Mitochondrial Ca^2+^ uniporter, Mitochondrial retrograde signaling, UPR^mt^, Intercompartmental proteostasis

## Abstract

Cross-compartment communication is critical for maintaining cellular homeostasis, which is essential for cell function and survival under stressful conditions. However, the cellular cues that trigger interorganellar communication remain poorly understood. Mitochondrial Ca^2+^ (_mt_Ca^2+^) homeostasis is fundamental to mitochondrial function; yet, how mitochondrial Ca^2+^ (_mt_Ca^2+^) homeostasis modulates nuclear gene expression to establish and maintain cellular homeostasis remains unclear. Here, we first characterize the critical role of the mitochondrial Ca^2+^ uniporter (MCU) in control of _mt_Ca^2+^ uptake and maintaining _mt_Ca^2+^ homeostasis *in planta.* Using gain-of-function and sextuple MCU knockdown mutants, we then analyzed the effects of impaired MCU-controlled _mt_Ca^2+^ homeostasis (iMUCH). We find that iMUCH elicits an interorganellar transcription program that activates multiple compartment-specific unfolded protein responses (UPRs) and genes critical for mitochondrial and cytosolic proteostasis. Additionally, iMUCH induces a post-transcriptional program that selectively represses the synthesis of ribosomes and RNA modification proteins. Furthermore, eukaryotic initiation factor α (eIFα and its phosphorylation likely serve as a protective mechanism under long-term mitochondrial proteotoxic stress induced by iMUCH. Collectively, the data demonstrate that MCU-controlled _mt_Ca^2+^ homeostasis plays a pivotal role in sustaining mitochondrial and cytosolic proteostasis through an interconnected organelle quality control system, which ultimately determines cell growth and fitness.

**Supplementary Information:**

The online version contains supplementary material available at 10.1007/s44154-026-00314-4.

## Introduction

Mitochondria are multifunctional organelles that host key biochemical pathways for energy conversion, as well as amino acid and lipid metabolism. Mitochondria also play a critical signaling role in cell growth, aging (senescence), programmed cell death (PCD), and stress responses by directly perceiving external stress stimuli, including extreme temperatures, drought, and high salinity, which adversely influence mitochondrial function and trigger mitochondrial stress (Van Aken et al. 2009; Zhu 2016). To cope with these stress challenges, mitochondria are in constant communication with the nucleus and the cytosol to reprogram nuclear transcription and to reset the cytosolic translation machinery, which are well characterized by the mitochondria-to-nucleus communication (Butow & Avadhani 2004; Quiros et al. 2016; de Souza et al. 2017; Andreasson et al. 2019; Mottis et al. 2019) and the mitochondrial-to-cytosolic response (Wang & Chen 2015; Wrobel et al. 2015; Kim et al. 2016; D'Amico et al. 2017; Boos et al. 2019). In animal cells, an imbalance in protein translation between nucleus-encoded and mitochondrion-encoded proteins often activates the mitochondrial unfolded protein response (UPR^mt^), which is a key mitochondrial retrograde signaling pathway that ensures proper translation, folding, and degradation of mitochondrion-localized proteins (Hartl et al. 2011; Houtkooper et al. 2013; Horwich 2014; Munch & Harper 2016). Whether these mitochondrial stress adaptive responses are conserved in plants and, if so, whether they share common players and biological consequences with their animal counterparts remains unclear.

The mitochondrial matrix Ca^2+^ (_mt_Ca^2+^) fluctuates in response to environmental and/or cellular cues (Logan & Knight 2003; Resentini et al. 2021). Disruption of _mt_Ca^2+^ homeostasis sensitizes mitochondria to the challenges of stress, aging (senescence), and apoptosis (Bernardi & Rasola 2007; Giacomello et al. 2007; Griffiths & Rutter 2009; Giorgio et al. 2018; Calvo-Rodriguez et al. 2020; Garbincius & Elrod 2022). However, it is less well understood how disturbed _mit_Ca^2+^ homeostasis impairs mitochondrial function, though it is acknowledged that mitochondrial Ca^2+^ uptake buffers cytosolic Ca^2+^ signaling (Rizzuto et al. 2012; Resentini et al. 2021). The mitochondrial Ca^2+^ uniporter protein complex (MCUC) in mammalian systems facilitates Ca^2+^ uptake through pore-forming MCUs whose activity is modulated by the regulatory components MITOCHONDRIAL Ca^2+^ UPTAKE (MICU) and ESSENTIAL MCU REGULATOR (EMRE) (Baughman et al. 2011; De Stefani et al. 2011; Hoffman et al. 2013; Sancak et al. 2013; Kamer & Mootha 2014; Wang et al. 2019; Liu et al. 2020). Structural analysis of MCUC by cryo-electron microscopy revealed the gating mechanism by which MICUs control uniporter activity (Fan et al. 2020). The plant MICU also negatively fine-tunes MCU-mediated mitochondrial Ca^2+^ uptake, thus preserving _mt_Ca^2+^ homeostasis (Wagner et al. 2015). Six putative *Arabidopsis thaliana* (Arabidopsis) MCU orthologs have been identified, each with a relatively conserved transmembrane domain, a pore loop, and the signature sequence DVME (Teardo et al. 2017). Arabidopsis MCU1, MCU2, MCU3, and MCU5 are localized to mitochondria, while MCU6 (CMCU) is targeted to both mitochondria and chloroplasts (Carraretto et al. 2016; Teardo et al. 2017, 2019; Selles et al. 2018). Stress stimulus-specific Ca^2+^ dynamics in the chloroplast stroma (_cs_Ca^2+^) are correlated with the transcript levels of *MCU6 *(Teardo et al. 2019). A recent study reported that mitochondrial Ca^2+^ uptake became limiting in the root cell of the *mcu1 mcu2 mcu3* triple mutant, demonstrating that MCU proteins mediate mitochondrial Ca^2+^ uptake in vivo (Ruberti et al. 2022).

Here, we generated sextuple MCU knockdown mutants and stable transgenic plants overexpressing MCUs and discovered that impaired MCU-controlled _mt_Ca^2+^ homeostasis due to gain-of-function and knockdown-of-function MCUs activates an interorganellar proteostasis transcription program that is counteracted in part by post-transcriptional repression. Collectively, our data demonstrate that _mt_Ca^2+^ homeostasis and cellular compartmental proteostasis are closely coordinated and determine cell growth and fitness.

## Results

### Mitochondrial Ca^2+^ uniporters are necessary for _mt_Ca^2+^ homeostasis in plants

**Fig. 1:**
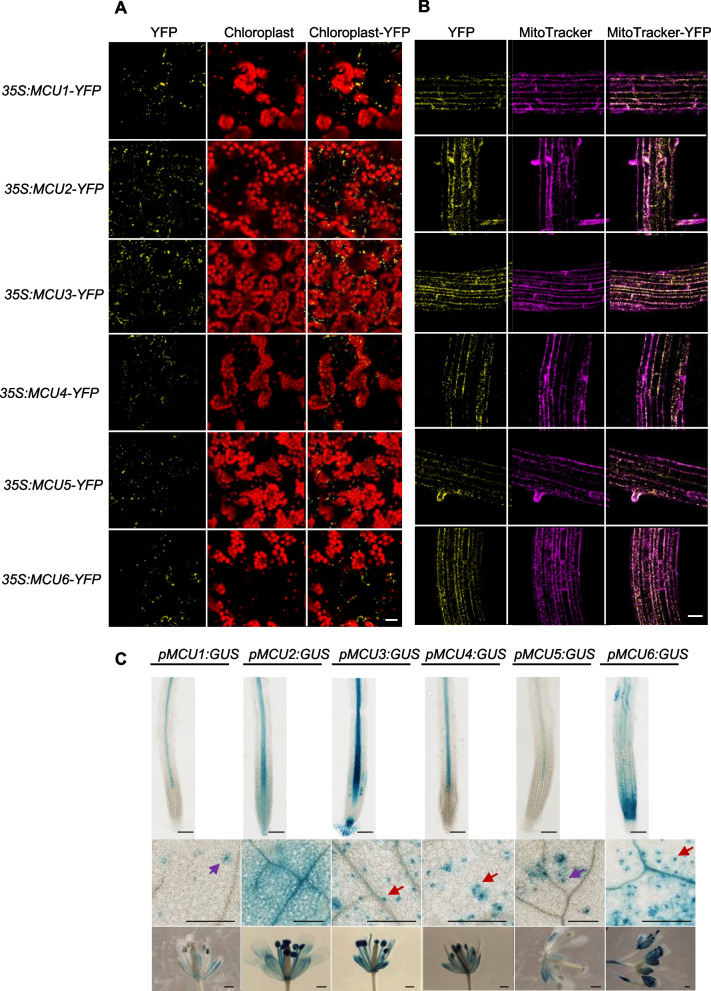
Arabidopsis MCU proteins localize to mitochondria across the tissues. **A** YFP signals in the leaf epidermal and mesophyll cells of stable Arabidopsis transgenic plants expressing *35S:MCU-YFP* as indicated. Autofluorescence of chlorophyll is shown in red. Scale bars, 20 µm. **B** YFP signals in root cells of stable Arabidopsis transgenic plants expressing *35S:MCU-YFP* as indicated. The root was stained with MitoTracker™ Deep Red FM (Invitrogen M22426). YFP signals in root cells are merged with the red signals of MitoTracker. Scale bars, 20 µm. **C** GUS-staining highlights tissue expression of MCUs. Expression patterns of MCU genes are shown in roots (upper panel), leaves (middle panel), and flowers (lower panel). Red arrows indicate guard cells, and purple arrows indicate trichomes. Scale bars, 0.5 mm

We first examined the subcellular localization of six putative MCUs (Teardo et al. 2017) in stable transgenic Arabidopsis plants (*35S:MCU1-YFP* to *35S:MCU6-YFP*). YFP signal was predominantly observed in mobile mitochondrion-like structures in epidermal cells and mesophyll cells of the leaf (Fig. 1A) and co-localized with the MitoTracker in root cells of these transgenic plants (Fig. 1B), supporting MCUs localization to the mitochondria. GUS staining showed that the 6 *MCU* genes were all expressed in the stele of the root, and *MCU3* was highly expressed in the root apex, including the root cap and quiescent center, and in the root cortex. In contrast, *MCU6* was highly expressed in the basal meristem (Fig. 1C, upper panel). *MCU3*, *MCU4*, and *MCU6*, but not *MCU1*, *MCU2*, and *MCU5*, were expressed in guard cells (Fig. 1C, middle panel). All six *MCU* genes were expressed in anthers; however, *MCU1* and *MCU5* expression was relatively low compared to the other 4 MCUs (Fig. 1C, lower panel). These results indicate that the six putative Arabidopsis MCUs are mitochondrial proteins with tissue-specific expression patterns.

Stress stimuli evoke cytosolic Ca^2+^ transient and mitochondrial Ca^2+^ uptake in plants (Knight et al. 1997; Logan & Knight 2003; McAinsh & Pittman 2009; Loro et al. 2012; Zhu et al. 2013; Carraretto et al. 2016; Teardo et al. 2019; Resentini et al. 2021; Ruberti et al. 2022). To examine the function of MCUs in the control of _mt_Ca^2+^ homeostasis, we generated transgenic Arabidopsis Col-0 lines in which Ca^2+^ reporter *Aequorin* was targeted to the mitochondrial matrix under the control of the ubiquitin promoter (Col:mtAq). We also constructed an *mcu*^*1−6*^ sextuple mutant by crossing *mcu* single mutants (Materials and Methods, Fig. S1A, B) to minimize functional compensation given the extensive functional redundancy within the plant *MCU* gene family. Then, we expressed the *mtAq* reporter construct into an *mcu* sextuple mutant to obtain *mcu*^*1−6*^*:mtAq*. RT-qPCR shows that all six *MCU* genes exhibit reduced expression in *mcu*^*1−6*^ sextuple mutant and *mcu*^*1−6*^*:mtAq* line, although the magnitude of reduction varies (Fig. S1C, D). For *MCU1*, *MCU3* and *MCU4*, T-DNA insertion in the exon is expected to produce a nonfunctional truncated protein. For *MCU2* and *MCU6*, insertions in promoter regions led to milder downregulation (less than 1.5-fold for *MCU2*, more than twofold for *MCU6*) (Fig. S1C and 1D). Given the residual transcript levels in several paralogs, *mcu*^*1−6*^ is a sextuple MCU knockdown mutant. To examine how overexpression of MCU could affect _mt_Ca^2+^ homeostasis, we chose one of the *MCU2* overexpression lines, *2OX-1,* which showed a high *MCU2* expression level compared to other overexpression lines of *MCU2* and MCU paralogs (Fig. 7C, Fig. S2) to ensure the effectiveness of the gain-of-function MCU. The *2OX-1* line was crossed with Col:*mtAq* to generate the *2OX:mtAq* line. Transcript analysis revealed a ninefold increased expression of *MCU2* in *2OX:mtAq* (Fig. S1D).

**Fig. 2:**
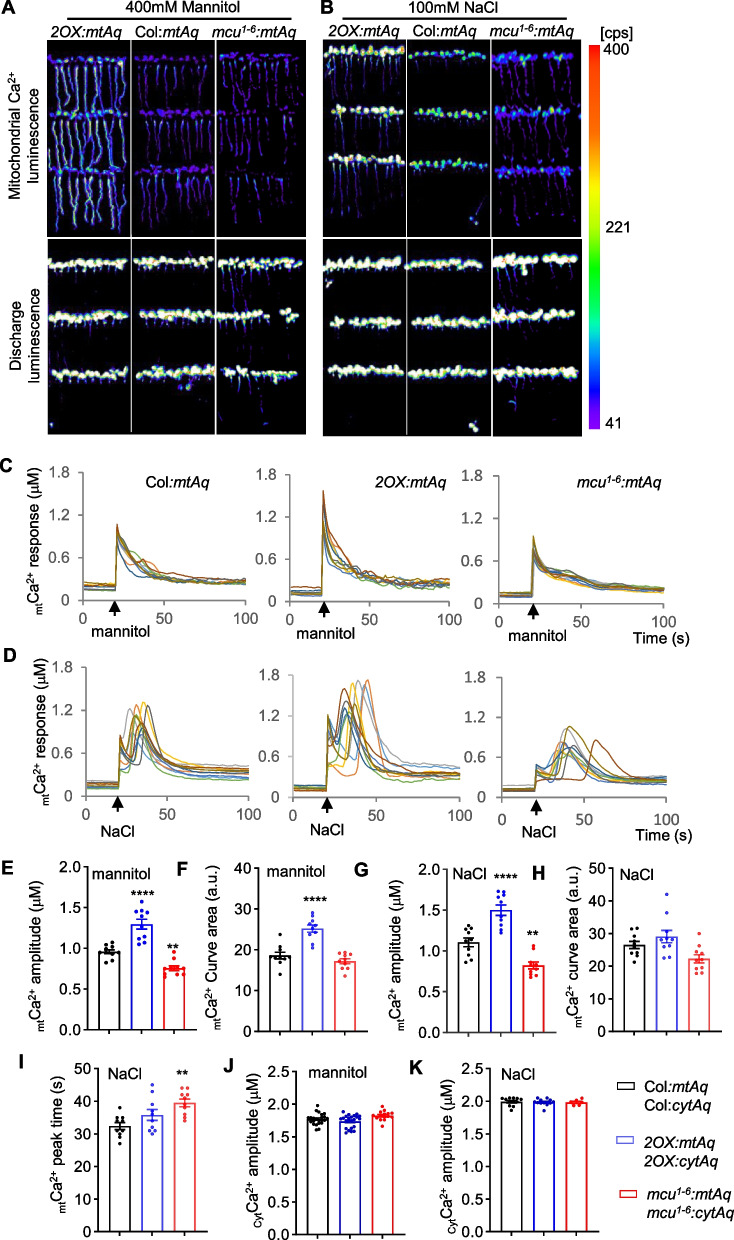
Altered *MCU*s expression affects _mt_Ca^2+^ responses. **A** and **B** FAS imaging of Aequorin-based _mt_Ca^2+^ luminescence. The _mt_Ca^2+^ response of *2OX:mtAq* and *mcu*^*1−6*^*:mtAq* mutant seedlings to 400 mM mannitol (A) or 100 mM NaCl (B) treatment is altered compared to wild type seedlings Col*:mtAq*, respectively. Upper panels are Ca^2+^ luminescence images for demonstrating maximum _mt_Ca^2+^ response, and lower panels are discharge luminescence images for counting total aequorin (see methods for details). FAS imaging was performed with more than six times, and representative images are shown. **C** and **D** Luminometer reading of Aequorin-based _mt_Ca^2+^ luminescence. The _mt_Ca^2+^ dynamic responses in Col*:mtAq, 2OX:mtAq* and *mcu*^*1−6*^*:mtAq* mutant seedlings treated with 400 mM mannitol (C) or 100 mM NaCl (D). Luminescence intensity was recorded at 1-s intervals, and the arrow indicates the starting point of treatment. Each colored line represents the _mt_Ca^2+^ dynamic response from one of 10 individual seedlings. **E** and **F** Signature of _mt_Ca^2+^ response to mannitol. The _mt_Ca^2+^ peak amplitude (E) and curve area (F) were quantified in 10 individual Col*:mtAq, 2OX:mtAq* and *mcu*^*1−6*^*:mtAq* mutant seedlings treated with 400 mM mannitol, respectively. **G-I** Signature of _mt_Ca^2+^ response to NaCl. The _mt_Ca^2+^ peak amplitude (G), curve area (H) and peak time (I) were quantified in 10 individual Col*:mtAq, 2OX:mtAq* and *mcu*^*1−6*^*:mtAq* mutant seedlings treated with NaCl, respectively. The _mt_Ca^2+^ luminescence peak value was determined by luminometer and converted to _mt_Ca^2+^ concentration as _mt_Ca^2+^ amplitude (see methods for details). The _mt_Ca^2+^ curve area is the normalized luminescence intensity determined by GraphPad Prism 9. A.u. stands for arbitrary unit. **J** and** K** Cytosolic Ca^2+^ responses are not altered in *2OX:cytAq* and *mcu*^*1−6*^*:cytAq* mutant seedlings. Cytosolic Ca^2+^ responses were recorded by FAS imaging, and *2OX:cytAq* and *mcu*^*1−6*^*:cytAq* mutant seedlings show no significant differences from Col*:cytAq* wild type seedlings in response to 400 mM mannitol (J) or 100 mM NaCl (K) treatment. Luminescence intensity of each FAS film containing about 30 seedlings was determined by indiGO™ software and converted to Ca^2+^ concentration (see methods). Results are expressed as mean ± SEM (*n* = 10 for E-I, *n* > 6 for J and K). Significant differences were examined from one-way ANOVA followed by Dunnett's multiple comparisons test, *P < 0.05, **P < 0.01. ***P < 0.001, ****P < 0.0001. See also Supplementary Table S7 for the information on statistical analysis

We used *2OX:mtAq* and *mcu*^*1−6*^*:mtAq* to determine the effect of overexpression and reduced expression of *MCU* on mitochondrial Ca^2+^ uptake in response to stimuli using Film Adhesive Seedling (FAS) imaging (Zhu et al. 2013) and luminometer reading. FAS imaging demonstrates that the _mt_Ca^2+^ in roots and leaves of *2OX:mtAq* seedlings showed a strong response to mannitol and NaCl but a relatively weak response in *mcu*^*1−6*^*:mtAq* compared to that of Col:*mtAq*, respectively (Fig. 2A and B). Although similar _mt_Ca^2+^ dynamics of *2OX:mtAq* and *mcu*^*1−6*^-*mtAq* were recorded by the luminometer (Fig. 2C and D), the higher _mt_Ca^2+^ amplitudes and curve area were detected in *2OX:mtAq* compared to Col:*mtAq* plants in response to mannitol and NaCl (Fig. 2E-H), indicating that the mitochondrial Ca^2+^ uptake is increased by overexpression of *MCU2*. By contrast, the *mcu*^*1−6*^*:mtAq* displayed a significant reduction in mannitol- and NaCl-triggered _mt_Ca^2+^ amplitudes, an average reduction of 21% and 26% for mannitol and NaCl, respectively (Fig. 2E-G), and a slight reduction in curve area compared to Col:*mtAq* plants (Fig. 2F and H). Both *2OX:mtAq* and *mcu*^*1−6*^*:mtAq* plants showed a delay in the peak time of _mt_Ca^2+^ response to NaCl (Fig. 2i). We observed no significant differences in the changes of cytosolic Ca^2+^ amplitudes between *2OX:cytAq* and Col:*cytAq* seedlings or between *mcu*^*1−6*^*:cytAq* and Col:*cytAq* seedlings in response to mannitol and NaCl (Fig. 2J and K). These results indicate that Arabidopsis MCUs play a role in the control of mitochondrial Ca^2+^ uptake for maintaining _mt_Ca^2+^ homeostasis.

### Impaired MCU-controlled _mt_Ca^2+^ homeostasis activates expression of nuclear genes important for mitochondrial proteostasis

**Fig. 3:**
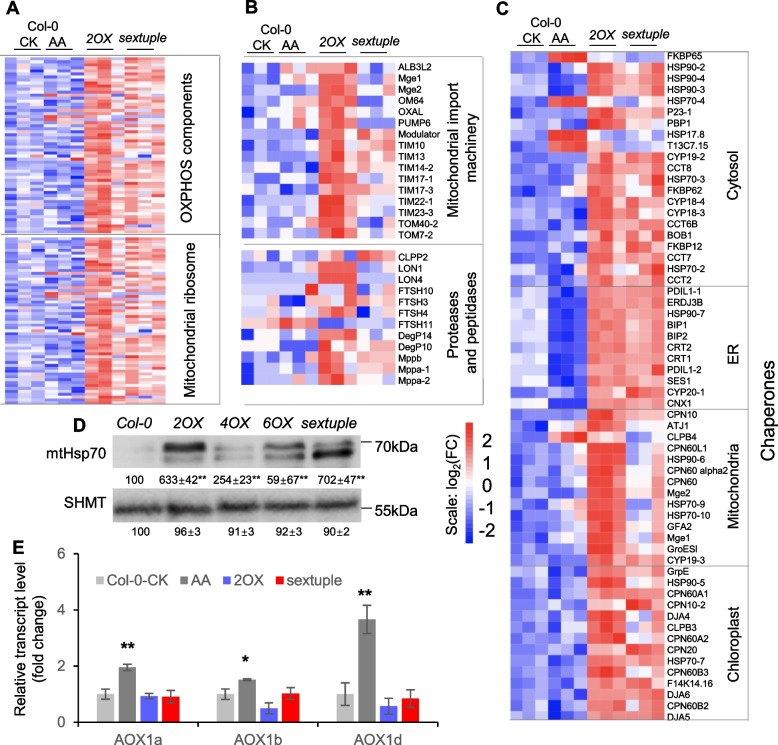
Transcriptional activation of the genes involved in control of mitochondrial proteostasis and multi-compartment UPRs in both gain-of-function and knockdown-of-function *MCU* plants. **A-C** Heatmap representation of expression of the nuclear genes encoding OXPHOS components and mitochondrial ribosomes (A), mitochondrial protein import machinery and proteases and peptidases (B), and chaperones of the cytosol, the ER, the mitochondria, and the chloroplast in AA-untreated and treated wild type, and in *2OX* and *sextuple* plants (C). Gene names are indicated on the right, and see also Dataset S2 for the list of gene IDs. **D** Comparison of mtHsp protein abundance. Western blots show induction of nDNA-encoded mtHsp70 proteins in 2OX, 4OX, 6OX and sextuple mutant plants. Mitochondrial matrix protein, SHMT is a loading control of total mitochondrial proteins. Quantifications of signals relative to the wild type (set to 100%) are provided below each panel as means ± SEM (*n* = 3). A two-tailed Student’s *t*-test was performed to examine statistical significance, *P < 0.05, **P < 0.01, with the fold change (vs wild type) |FC|> 1.2. **E** Comparison of the expression of the genes in the alternative respiration pathway. A two-tailed Student’s *t*-test was performed to examine statistical significance, *P < 0.05, **P < 0.01. See also Supplementary Table S7 for the information on statistical analysis

We assessed whether impaired MCU-controlled _mt_Ca^2+^ Homeostasis (iMUCH) due to gain-of-function and knockdown-of-function *MCU* may evoke an undefined retrograde signaling pathway communicating with nuclei. It is known that antimycin A (AA, an electron transport chain inhibitor) causes mitochondrial stress and triggers mitochondrial retrograde response (MRR) in plants (Wikstrom & Berden 1972; Slater 1973; Huang et al. 2005; Ng et al. 2013; Ivanova et al. 2014; Zhao et al. 2025). To compare the effects of iMCUH and AA treatment on nuclear gene expression, we performed RNA-seq analysis and identified 2,282, 482, and 3,864 differentially expressed genes (DEGs, |FC|> 1.5 and padj < 0.05) in *2OX* and sextuple mutant and in AA-treated wild type, respectively (Supplementary Table S1). Interestingly, an activation of the genes for mitochondrial proteostasis, including the mitoribosome, the oxidative phosphorylation (OXPHOS) complexes, mitochondrial protein import, and proteases, was evident in both *2OX* and the sextuple mutant but not in AA-treated wild type (Fig. 3A and B). Moreover, a group of chaperone genes encoding mitochondrial (mtHsp), cytosolic (cyHsp), endoplasmic reticulum (ER) (erHsp), and chloroplast (cpHsp) chaperones were also induced in *2OX* and the sextuple mutant compared to the wild type, but the majority of such inductions were not found in AA-treated plants (Fig. 3C, Supplementary Table S2). These results indicate that multiple compartmental UPRs in addition to the UPR^mt^ are activated in both *2OX* and the sextuple mutant, although some of them are less pronounced in the sextuple mutant. A similar transcriptional response was not detected in AA-treated plants. A high abundance of mitochondrial chaperone mtHsp70 protein is indeed present in 2OX and sextuple mutant, as well as in other *MCU* overexpression lines (Fig. 3D, Fig. S2). However, the AA-induced alternative respiration pathway was not detected in *2OX* and the sextuple mutant, as the expression levels of AOX genes remained unchanged (Fig. 3E).

Together, these analyses revealed that iMUCH activates a transcription program that regulates expression of the nuclear genes important for mitochondrial proteostasis, which is different from AA-induced MRR. Activation of multi-compartment *UPR* reflects the potential significance of mitochondrial cross-compartmental signaling in maintaining cellular proteostasis upon iMUCH.

### Post-transcriptional repression counteracts iMUCH-evoked transcriptional activation of cytosolic ribosomal gene expression

**Fig. 4:**
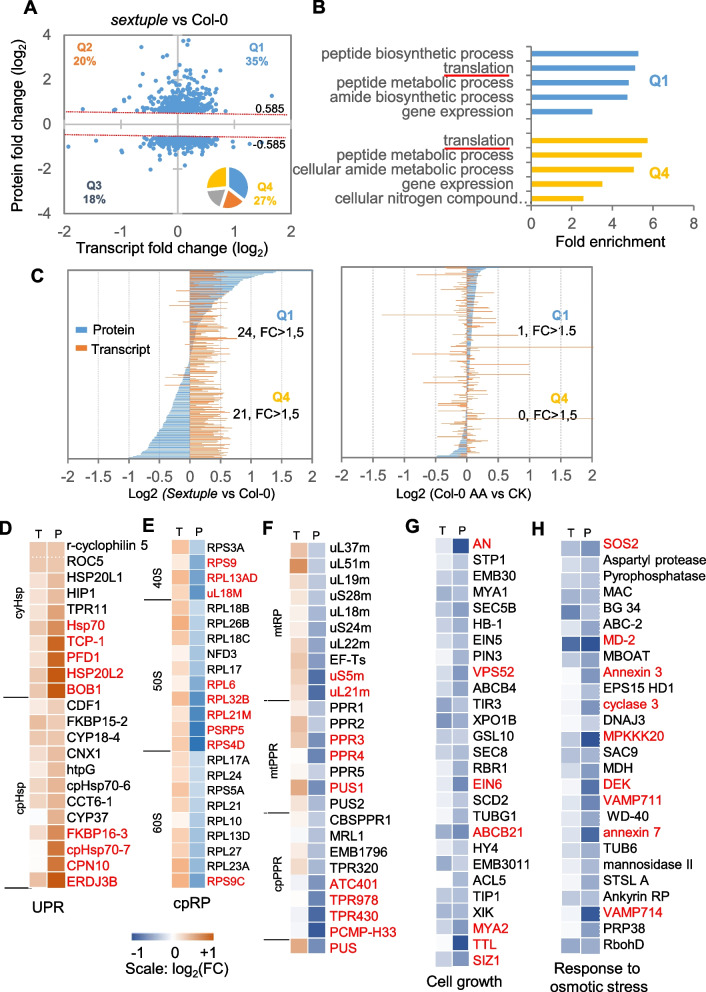
Proteome–transcriptome cross-comparison reveals post-transcriptional repression of ribosome gene expression. **A** Co-regulation plot of transcript and protein fold-change (FC) of sextuple mutant seedlings versus wild type seedlings. The percentage of co-upregulated proteins (Q1), post-transcriptionally increased proteins (Q2), co-downregulated proteins (Q3), and post-transcriptionally repressed proteins (Q4) is indicated in each quadrant. **B** Top five biological processes enriched in Q1 and Q4 quadrant of the co-regulation plot, derived from the significant hits of differentially abundant proteins (DAPs, *P* < 0.05, |FC|> 1.5). **C** Co-regulation of cyRPs. Individual transcripts (yellow) and proteins (blue) were plotted with Log_2_(FC) of sextuple mutant seedlings versus wild type seedlings (left panel) and AA-treated seedlings versus untreated seedlings (right panel), demonstrating a large proportion of post-transcriptionally repressed cyRPs in sextuple *mutant* seedlings, but not in AA-treated seedlings. **D**-**H** Examples of co-regulated proteins identified by mass spectrometry and RNA-seq. Co-upregulated chaperones in Q1 are transcriptionally and post-transcriptionally induced (D). nDNA-encoded cpRPs (E), mtRPs, mtPPRs, and cpPPRs (F) in Q4 are transcriptionally induced, but post-transcriptionally repressed. An example of co-downregulated proteins in cell growth (G) and response to osmotic stress (H) is also demonstrated. Proteins marked by the red color are the ones with |FC|> 1.5. *P* < 0.05. See also Supplementary Table S4 for the information on gene lists for heatmap analysis

To better understand how iMUCH evoked transcriptional response determines the proteome, we performed a mass spectrometry analysis of the proteome and identified 694 and 11 differentially abundant proteins (DAPs, |FC|> 1.5 and *p*-value < 0.05) in the sextuple mutant and in AA-treated wild type, respectively (Supplementary Table S3). To examine how the changes in transcript and protein levels were correlated in cellular processes, we performed a co-regulation analysis by plotting the FC of DAPs versus the FC of transcript levels for 681 protein–transcript pairs with the above criteria applied to proteins (|FC|> 1.5, *p*-value < 0.05) and no criteria applied to transcripts (Fig. 4A; Supplementary Table S4). Co-regulation analysis revealed that about half of all genes were coordinately regulated, while the remaining half were regulated differently at the protein and transcript levels in the sextuple mutant (Fig. 4A). Genes with upregulated transcripts and upregulated (Q1) or downregulated proteins (Q4) showed enrichments for translation (Fig. 4B). Manual inspection of ribosome (*RP*) genes involved in cytosolic translation revealed that 104 out of 194 cytosolic RPs (cyRPs) are altered in their abundance, of which 42% belong to Q1 and 58% to Q4, indicating that about half of all *cyRP* genes are repressed post-transcriptionally (Fig. 4C; Supplementary Table S4). No cyRPs were detected as DAPs in AA-treated wild type (Fig. 4C; Supplementary Table S3). In the manual inspection of Q1, we found 10 cytosolic and 12 chloroplast proteins in protein folding (GO:000645), most of which are increased with FC > 1.5 (Fig. 4D). Q4 includes most nDNA chloroplast (*cpRP*s), mitochondrial (*mtRP*s) ribosome proteins, and mitochondrion- and chloroplast-localized pentatricopeptide repeat proteins (PPRs), tetratricopeptide repeat proteins (TPRs), and pseudouridine synthases (PUSs) involved in organellar RNA editing and translation (Fig. 4F). These results indicate that a large fraction of cytosolic and organellar ribosome proteins, as well as proteins with roles in cytosolic and organellar RNA editing and translation, are subjected to post-transcriptional repression. Fewer proteins were identified in Q2 and Q3, but “cell growth (GO:0016049)” and “response to osmotic stress (GO:0006970)” were enriched in Q3 with DAPs, with filtering criteria of |FC|> 1.2 and *p*-value < 0.05, among which some proteins were reduced with FC > 1.5 (Fig. 3G and H). These results indicate that a post-transcriptional repression of cytosolic and organellar ribosome proteins and cytosolic and organellar RNA editing proteins and a transcriptional and post-transcriptional co-repression of growth-related genes and stress-responsive genes occur upon iMUCH.

### Selective translational repression of ribosomal and RNA modification genes is evident in both gain-of-function and knockdown-of-function *MCU* plants

**Fig. 5:**
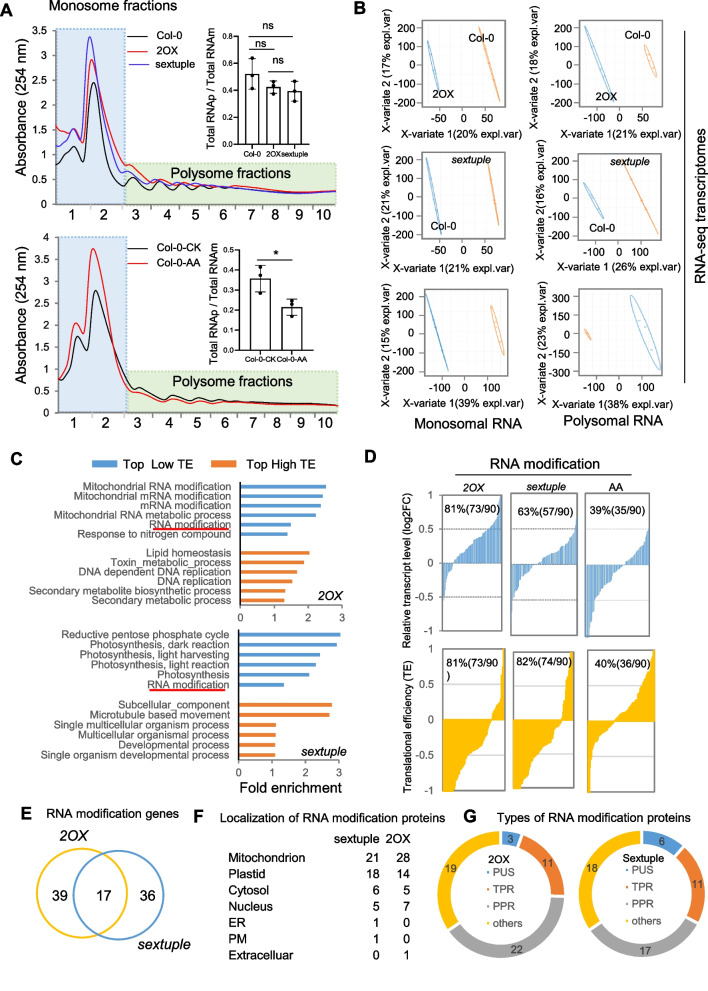
*2OX* and *sextuple* plants display reduced translational efficiency of RNA modification proteins and cytosolic ribosomal proteins. **A** Representative polysome profiles showing altered cytosolic polysome abundance in *2OX* (red line) and sextuple mutant (blue line) seedlings relative to the wild type (black line) (upper panel) and in AA-treated (red line) and control (black line) wild type seedlings (lower panel). Monosomal fractions and polysomal fractions are indicated by the blue and green backgrounds, respectively. RNAs isolated from highly translated fractions (green, polysomes) and lesser translated fractions (blue, monosomes) were used for RNA-seq analysis for *2OX*, sextuple mutant, and wild type seedlings as well as AA-treated and control wild type seedlings. The inserted panels are the histogram of total polysomal RNA (RNAp) divided by total monosomal RNA of 2OX, sextuple mutant and wild type plants (upper insertion), and that of AA-treated and untreated plants (lower insertion). A two-tailed Student’s *t*-test was performed to examine statistical significance, *P < 0.05, ns: not significant. Partial least-squares discriminant analysis (PLS-DA) for monosomal (left) and polysomal (right) RNA-seq libraries showing the distinction between *2OX* (upper panel), sextuple mutant (middle panel) seedlings and their controls and between AA-treated and control wild type seedlings (lower panel). **C** Top six biological processes with highest enrichment scores for low translational efficiency (TE, blue) or high TE (yellow) transcripts in GO. TE is defined as the Log_2_ ratio of polysomal versus monosomal differences in transcript levels between *MCU* and control seedlings or AA-treated and control wild type seedlings. Decreased TE was detected in pathways of mitochondrial RNA and RNA modification, reductive pentose phosphate cycle, and photosynthesis in *2OX* and sextuple mutant seedlings, but not in AA-treated seedlings (Fig. S5). Clusters with an “enrichment score” above 1 (*P* < 0.001) were considered significantly enriched. **D** Visualization of transcript and TE levels of mRNAs enriched in RNA modification. Proportion of downregulated and upregulated transcripts (upper panel) and their TEs (lower panel) were plotted for *2OX*, sextuple mutant, and AA-treated seedlings relative to their controls. The percentage of upregulated transcripts and transcripts with low TEs is indicated within each plot. **E**–**G**. Identity of genes enriched in RNA modification. The Venn diagram shows the number of overlapping genes and unique genes detected in *2OX* and sextuple mutant seedlings, respectively (E), with their predicted subcellular localization (F), and the number of genes encoding PPR-related proteins in both *2OX* and sextuple mutant seedlings (G). See also Supplementary Table S5 for the information on lists of genes for GO analysis

Post-transcriptional repression of ribosome gene expression suggests a cytosolic proteostatic response may occur upon iMUCH. To test this possibility, we performed polysome profiling in *2OX*, sextuple, and wild type to assess the status of cytosolic translation (Molenaars et al. 2020). We observed a slight shift from polysomes to monosomes in *2OX* and sextuple mutant plants relative to the wild type (Fig. 5A, upper panel) but a significant shift in AA-treated wild type (Fig. 5A, lower panel). These observations suggest that impairing electron transport chain (ETC) function, but not iMCUH, significantly represses global translation.

To determine which genes are regulated translationally, we generated RNA-seq libraries of the monosomal and polysomal RNA fractions. This RNA-seq dataset provided information for over 23,000 genes, covering the majority of the Arabidopsis genome and allowing for a global description of the differences between monosomal and polysomal fractions (Supplementary Table S5). We performed a partial least-squares discriminant analysis (PLS-DA) to distinguish *2OX* and the sextuple mutant from wild type and AA-treated and untreated wild type (Fig. 5B). Next, we calculated the translational efficiency (TE) of each gene (see Methods). A positive log₂ TE value indicates a higher TE for a given transcript that shifts from monosomes to polysomes, whereas a negative log₂ TE value represents a lower TE of a given transcript that shifts from polysomes to monosomes. We applied a minimal fold-change TE cutoff of 1.25 with a significant Variable Importance in Projection (VIP) score to filter genes with significantly altered TE (saTE) for GO term clustering (Supplementary Table S5). Intriguingly, genes with low TE in both *2OX* and the sextuple mutant, but not in AA-treated wild type, are enriched in mitochondrial RNA metabolism and RNA modification (Fig. 5C), indicating that the selective translational repression of RNA modification genes takes place upon iMUCH. We inspected our RNA-seq data and determined that about 81%, 63%, and 39% of RNA modification genes were upregulated in *2OX*, the sextuple mutant, and AA-treated wild type, respectively. About 81% of their transcripts with low TE were detected in *2OX* and the sextuple mutant, but only 40% in AA-treated wild type, indicating again the high translational suppression of RNA modification genes in *2OX* and the sextuple mutant (Fig. 5D). Among the enriched RNA modification genes with low TE, we identified 56 (*2OX*) and 53 (sextuple mutant) genes as significant hits, of which 17 genes were shared by *2OX* and the sextuple mutant (Fig. 5E). Of those, about half encoded mitochondrion- or chloroplast-localized PPR, TPR, and PUS proteins, which regulate organellar functions by executing organellar RNA editing and translation (Fig. 5F and G) (Sun et al. 2019, 2024). Although *cyRP*s were not part of the top list of enriched genes with saTE, we also assessed their translational efficiency. About 88% and 93% of *cyRPs* were transcriptionally upregulated in *2OX* and the sextuple mutant, respectively (Fig. S3A, Supplementary Table S2). However, only about half (52%) of *cyRPs* were upregulated upon AA treatment (Fig. S3A, Supplementary Table S5). A large proportion of *cyRP*s had low TE in *2OX* (76% of cyRPs) and the sextuple mutant (62% of *cyRP*s), and 51% of *cyRP*s with low TE were detected in AA-treated wild type, implying a strong repression of cytosolic translation of *cyRP* genes in *2OX* and the sextuple mutant (Fig. S3B, Supplementary Table S5). Genes with a high TE encoded proteins in lipid homeostasis and secondary metabolism in 2OX and in protein transport and establishment of protein localization in the sextuple mutant (Fig. 5C, Supplementary Table S5). This observation suggests that metabolic adaptation and the enhancement of interorganellar contacts are likely activated upon iMUCH (Kim et al. 2016; Antonicka et al. 2020). Genes with high TE were enriched mostly in organic acid metabolism and response to oxygen level in AA-treated wild type (Fig. S4), indicating that translational adaptation mostly works towards metabolism and stress response to AA-induced mitochondrial stress.

These findings suggest that genes in organellar and cytosolic translational machinery are subjected to translational regulation upon iMUCH. Although the global translational response was less affected in *2OX* and the sextuple mutant compared to AA-treated wild type, a prominent translational repression of RNA modification and cytosolic ribosome proteins took place in 2*OX* and the sextuple mutant, but not in AA-treated wild type. These results clearly demonstrate that iMUCH strongly triggers a cytosolic translation response with specific impacts on RNA modification protein and cytosolic ribosome protein synthesis.

### eIF2α but not TOR responds to iMUCH-induced long-term mitochondrial stress

**Fig. 6:**
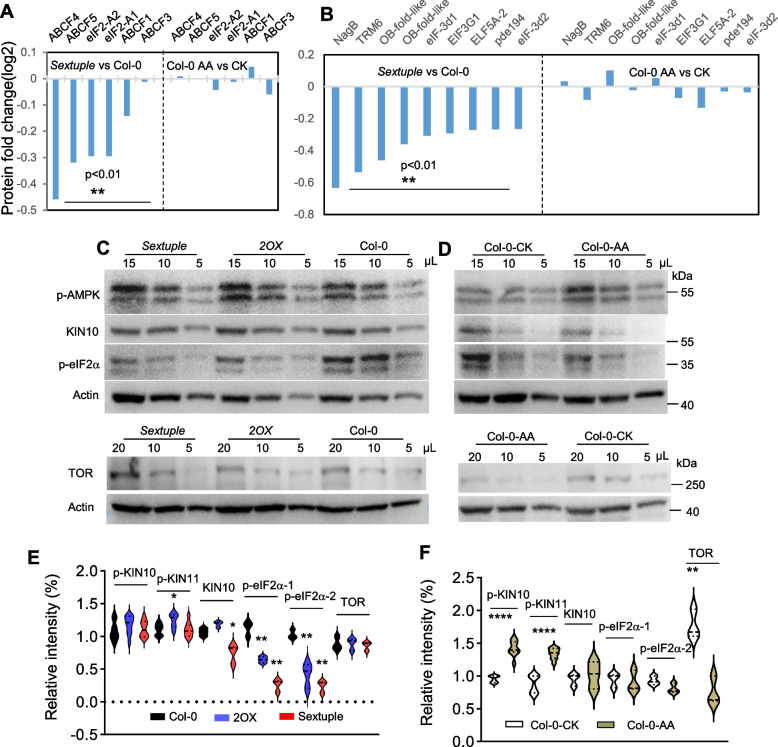
The eIF2α but not TOR responds to iMUCH. **A** and **B** protein abundance of components in GCN2-eIF2α pathway (A) and in translation initiation (GO:0006413) (B). Comparison of protein abundances between the sextuple mutant and Col-0, and between Col-0-AA and Col-0 controls was performed by quantitative proteomic analysis. **C** and **D** protein abundances of TOR and eIF2α pathway components were detected by immunoblotting. Samples and loading volumes are indicated at the top of the panel. Actin serves as a loading control. Anti-p-AMPK antibody recognizes p-KIN10 (upper band) and p-KIN11 (lower band). **E** and **F** quantification of protein abundance. Signal intensity of each protein was quantified with at least three immunoblots using Evolution-Capt software. Significant differences were examined with one-way ANOVA followed by Dunnett's multiple comparisons test for E, *P < 0.05, **P < 0.01, and ****P < 0.0001, *n* = 3. A two-tailed Student t-test was performed for F, ****P < 0.0001, *n* = 3. See also Supplementary Table S7 for the information on statistical analysis

Protein synthesis can be attenuated by the decrease in the Mechanistic Target of Rapamycin (mTOR) signaling activity and the increase in the phosphorylation of eukaryotic translation initiation factor 2 (eIF2α Samluk et al. 2019; Topf et al. 2019). To identify the potential effectors, we mined the proteomic data and discovered that eIF2α, eIF2-A2, ABCF4, and ABCF5 protein levels are significantly lower in the sextuple mutant but not in AA-treated wild type (Fig. 6A), although most TOR components were unfortunately not detected by mass spectrometry. Besides, other components in translation initiation also showed a significant reduction, including eIF3d (cytoplasmic cap-dependent translation initiation) and EIF3, TRM61 and NagB (regulation of translation initiation) (Fig. 6B). To substantiate these observations, we further examined the abundance of p-eIF2α phosphorylated form of eIF2α), KIN10, the phosphorylated form of KIN10 (p-KIN10), KIN11 (p-KIN11), and TOR protein by immunoblotting. The p-eIF2α levels were significantly reduced, but p-KIN10 and TOR abundance showed no obvious changes in *2OX* or the sextuple mutant, although a slight increase of p-KIN11 in *2OX* and a decrease of KIN10 in the sextuple mutant were detected (Fig. 6C, E). By contrast, we detected a significant increase in p-KIN10 and p-KIN11 and a reduction of TOR, but no obvious alteration of p-eIF2α abundance in AA-treated wild type, although KIN10 showed no changes in AA-treated wild type (Fig. 6D, F). These results suggest that the translational response to iMUCH and AA-induced mitochondrial stress may be transduced by different pathways.

### iMUCH is coupled with the reduction of growth and stress resistance

**Fig. 7:**
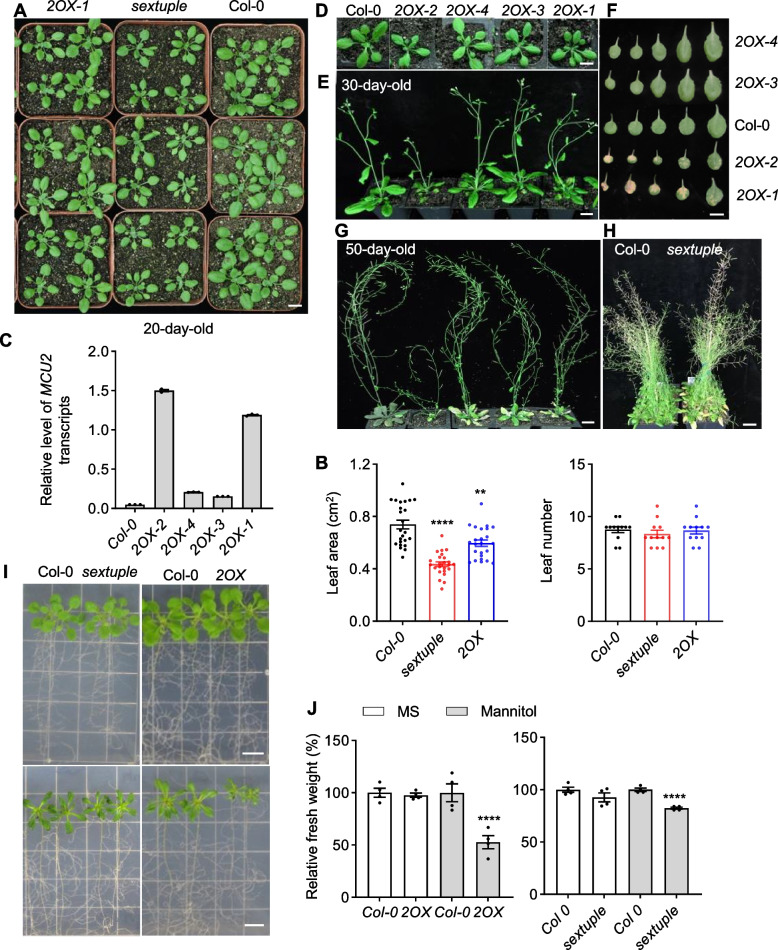
*2OX* and *sextuple* mutant show reduction in growth, osmotic stress resistance, and acceleration of leaf senescence. **A**, **B** Morphological comparison of seedlings grown in soil under normal growth conditions. The morphology of 10-day-old seedlings (A) and the area of the two largest rosette leaves and the leaf number of 10-day-old seedlings (B) were recorded. Results are shown as the mean ± SEM (*n* = 24). A two-tailed Student’s *t*-test was performed. **P < 0.01, ****P < 0.0001. **C** Relative transcript levels in *MCU2* transgenic lines measured by RT-qPCR. **D** The morphology of 10-day-old seedlings of *MCU2* transgenic lines. **E** The morphology of 30-day-old seedlings of *MCU2* transgenic lines. **F***2OX* plants show MCU2 expression level-dependent early leaf senescence. Rosette leaves of 30-day-old seedlings from 2OX-1 and 2OX-2 show premature yellowing starting from the lowest, older leaves, while leaves of Col-0, 2OX-3, and 2OX-4 remain green. **G** The morphology of 50-day-old seedlings of *MCU2* transgenic lines. **H** Sextuple mutant shows accelerated leaf senescence compared to wild type plants at the mature stage under normal growth conditions. Rosette leaves of 50-day-old seedlings from the sextuple mutant are fully yellowed, while those of Col-0 remain green. **I** 2OX and sextuple mutant are sensitive to mannitol-induced osmotic stress. Five-day-old seedlings were transferred to plates containing MS medium only (upper panel) or MS medium containing 150 mM mannitol (lower panel). Photographs were taken 10 days after transfer. **J** Relative fresh weight of *2OX* and sextuple seedlings compared to wild type, measured 10 days after transfer. Results are shown as the mean ± SEM (*n* = 4), normalized to wild type values (set to 100%). Significantly different levels were examined with a two-tailed Student’s *t*-test (****P < 0.0001). The scale bar is 1 cm. See also Supplementary Table S7 for the information on statistical analysis

The connection between mitochondrial and cytosolic proteostasis is likely related to organismal health and cellular stress resistance (Merkwirth et al. 2016; Wang & Auwerx 2017; Boos et al. 2020). To determine the physiological consequence of iMUCH-evoked proteostatic response, we thus examined the growth and stress resistance of the sextuple mutant and four independent *2OX* transgenic lines. We first tracked the seedling growth and development of four *2OX* lines and found that the size of *2OX* plants is correlated with MCU2 levels, indicating that growth inhibition is related to MCU2 expression (Fig. 7A-C). A significant reduction in the leaf area but not in the leaf number of *2OX* and the sextuple mutant was observed compared to the wild type (Fig. 7A and B). Differences in growth were minimized as the developmental process in the sextuple mutant. In addition, *2OX* lines and the sextuple mutant showed premature leaf senescence under normal growth conditions. The timing of premature leaf senescence also appeared to be *MCU2* level-dependent (Fig. 7C and D). However, as the plants matured, all *2OX* lines showed earlier leaf senescence compared to the wild type (Fig. 7D). The sextuple mutant exhibited premature leaf senescence in the late stage under normal growth conditions (Fig. 7D). We tested the resistance to mannitol-induced osmotic stress and found that the growth of *2OX* and *the sextuple* mutant was significantly inhibited compared to the wild type (Fig. 7E and F). These results show that iMUCH shapes plant growth, development, and stress resistance.

## Discussion

In our study, we observed mitochondrially localized MCUs in transgenic plants overexpressing MCUs, including MCU6, which was previously reported as a mitochondrial and chloroplast dual-localized Ca^2+^ uniporter when driven by the endogenous promoter (Teardo et al. 2019). It seems that constitutively expressed MCU6 is only targeted to mitochondria. Nevertheless, our results indicate that enhancing or impairing MCU functions caused specific changes in _mt_Ca^2+^ dynamics, further highlighting the important roles of MCUs as mitochondrial Ca^2+^ uptake systems in maintaining _mt_Ca^2+^ homeostasis and in sensing changing (micro)environments for mitochondrial function. Recent studies reported the significantly reduced _mt_Ca^2+^ basal level and the mitochondrial Ca^2+^ uptake in the root cell of the Arabidopsis *mcu1 2 3* triple mutant (Ruberti et al. 2022). The activity of mitochondrial Ca^2+^ uptake in the root cell matches the expression pattern of *MCUs* in the root cell. And the residual Ca^2+^ uptake activity in the *mcu1 2 3* line is likely due to the predominant expression of MCU6 in roots (Fig. 1C). In addition, we observed that MCU-mediated mitochondrial Ca^2+^ uptake does not appear to substantially affect _cyt_Ca^2+^ dynamics, suggesting that MCU-mediated mitochondrial Ca^2+^ uptake may have a limited capacity to buffer _cyt_Ca^2+^. Alternatively, lack of changes in _cyt_Ca^2+^ may be due to the limitation of our study in which _mt_Ca^2+^ signal responses were recorded at the whole plant level (Zhu et al. 2013). Thus, we cannot exclude the possibility that MCUs may buffer _cyt_Ca^2+^ within microdomains.

The physiological roles of organellar Ca^2+^ homeostasis in organelle functioning are largely unknown (Resentini et al. 2021). A recent study found that the jasmonic acid (JA)-related transcripts are significantly repressed in the *mcu1 2 3* triple mutant seedling root transcriptome and proposed that the deregulated JA homeostasis may account for the decreased touch response in the *mcu1 2 3* triple mutant (Ruberti et al. 2022). No DEGs are enriched in the JA signaling pathway in the 10-day-old seedling transcriptome of 2OX and sextuple mutant (Fig. S6, Supplementary Table S1), which is probably due to the tissue- and physiological stage-specific patterns of gene expression. Here, iMUCH activated a nuclear transcription program that is very similar to the Msn2/4-dependent interorganellar proteostasis transcription program (IPTP) induced by the deficiency in mitochondrial translation accuracy (Suhm et al. 2018). Thus, we assume that iMUCH may cause proteotoxic stress in the matrix of mitochondria, likely due to the disturbed mitochondrial translation. Although we do not have direct evidence, alterations in the protein abundance of OXPHOS complexes may be the outcome of disturbed mitochondrial translation. Our immunoblotting showed that the abundance of mtDNA-encoded COB, COX1, ATP4, and ATP8 is reduced in *2OX* and sextuple mutants, as well as in MCU4 (*4OX*) and MCU6 (*6OX*) overexpression plants compared to the wild type (Fig. S5A-5D), although complex I subunits NAD2 and NAD5 appeared less affected (Fig. S5A). nDNA-encoded subunits CA3 and COX X1 both increased (Fig. S5A, 5 C), while CYC1 and ATP2 remained unchanged (Fig. S5B, 5D). Therefore, an apparent stoichiometric imbalance between nDNA and mtDNA-encoded subunits was apparently evident for complexes lll, IV, and V.

**Fig. 8:**
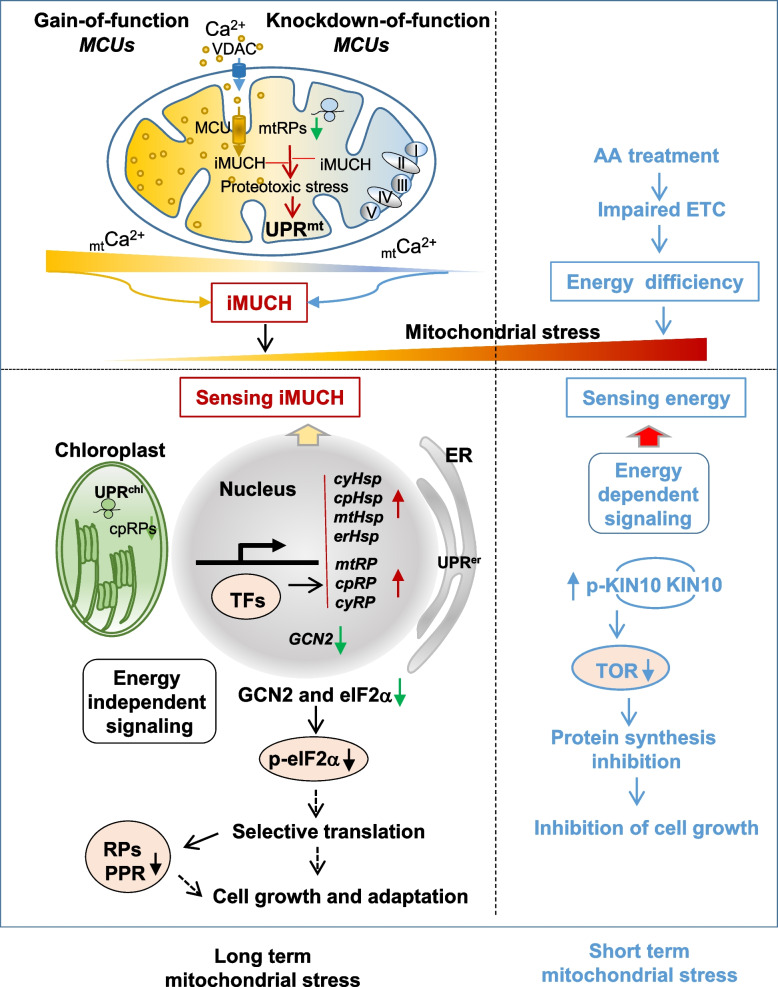
Perturbation of mitochondrial Ca^2+^ homeostasis activates a cross-compartmental proteostatic response. Gain-of-function (*2OX*) or knockdown-of-function (*sextuple* mutant) MCU proteins impairs MCU-controlled mitochondrial Ca^2+^ homeostasis (iMUCH). iMUCH disturbs mitochondrial translation that causes proteotoxic stress within mitochondrial matrix. The signals derived from iMUCH are relayed by unknown transcription factors (TFs) to the nucleus to activate a transcriptional program to restore mitochondrial proteostasis. A selective translational repression counteracts transcriptional activation in cytosol to establish cellular proteostasis. Reduction of eIF2α phosphorylation (p-eIF2α) is likely attributed to transcriptional and post-transcriptional co-suppression of GCN2, and selective translational repression of eIF2α. Reduction of eIF2α and its phosphorylation and selective synthesis of proteins are the part of adaptive mechanisms that protect the cell from severe inhibition of protein synthesis and maintain cellular proteostasis for redirecting cell growth and stress adaptation under iMCUH-induced long-term mitochondrial stress. AA-impaired respiration electron transfer chain (ETC) causes cellular energy deficiency, and evokes a short-term acute mitochondrial stress that is sensed by KIN10 through phosphorylation of KIN10 (p-KIN10). An increase of p-KIN10 inactivates TOR activity. An energy-dependent signalling pathway mediates AA inhibition of cell growth. Together, it is proposed that the GCN2-eIF2α and TOR-S6K pathways mediate mitochondrial stress-dependent regulation of protein synthesis by sensing iMUCH and cellular energy status, respectively. Dashed lines indicate the postulated processes, and solid lines indicate defined processes in this study

The consequence of a mitonuclear protein imbalance has not been fully explored yet in plants, although studies in other systems reveal that mitonuclear protein imbalances trigger a nuclear transcriptional response to ultimately restore mitochondrial proteostasis by activation of the UPR^mt^ (Houtkooper et al. 2013). And in plants, interrupting mitochondrial ribosome function was shown to affect mitochondrial gene expression by altering translation and splicing (Kwasniak et al. 2013; Kwasniak-Owczarek et al. 2019). Here, the iMUCH evoked a transcriptional response with most upregulated hits of nuclear genes important for mitochondrial proteostasis and cytosolic ribosomes, which are likely to restore mitochondrial proteostasis (Fig. 3A-D). However, transcriptional activation of cytosolic, mitochondrial, chloroplast, and ER chaperone genes highlights the cross-compartmental communication that is likely initiated by iMUCH-activated UPR^mt^ that is connected to the UPR response not only in the cytosol (UPR^cyt^), but also in the chloroplast (UPR^chl^) and ER (UPR^er^) (Fig. 8). These results suggest that an intrinsic cellular compartmental communication network operates when mitonuclear protein imbalance occurs for maintaining cellular homeostasis in plants. Intriguingly, this intrinsic communication network is also effective at the post-transcriptional level with a selective translational repression of nDNA *cyRP*s, *mtRP*s, and *cpRP*s, implying that signals derived from iMUCH are not only relayed to the nucleus but also to the cytosol for post-transcriptional regulation of intercompartmental proteostasis (Fig. 8). More than half of all transcriptionally induced *cyRP*s are translationally repressed, as evidenced by their reduced translational efficiency and protein abundance. While cyRPs control cytosolic proteostasis, RNA modification proteins likely are key players in tuning organellar proteostasis since these proteins function in organellar RNA editing and translation (Sun et al. 2019). Mitoribosomes contain 10 PPR proteins, and rPPR1 is a generic translation factor for mitochondrial translation (Waltz et al. 2019; Sun et al. 2024). Although rPPR1 was not detected in our proteomic analysis, rPPR9 (At1g60960) was significantly reduced in the sextuple mutant, but not in AA-treated wild type plants (Supplementary Table S5). These results imply that both RPs and PPR proteins are effectors in iMUCH-evoked interorganellar proteostatic response. Therefore, our findings provide a framework for understanding how a regulatory network integrates transcriptional and post-transcriptional mechanisms to coordinate nuclear-encoded gene expression and maintain cellular homeostasis (Couvillion et al. 2016; St-Pierre & Topisirovic 2016).

Signals and their transducers in iMUCH-evoked interorganellar proteostatic responses are unknown. Our data do not support the notion that reactive oxygen species (ROS) act as the primary signal for the initiation of the transcriptional response because *AOX1a* transcript levels were not significantly raised in *2OX and* sextuple mutants (Fig. 3E). Likewise, AA treatment induces *AOX1a* expression, but it cannot elicit the same response as iMUCH. Phosphorylation of eIF2α has been implicated in translational repression in response to various stresses (Wang et al. 2017; Lokdarshi et al. 2020). We found that *eIF2*α was transcriptionally upregulated, but the level of eIF2α protein was significantly reduced; thus, *eIF2*α is also subjected to selective translational repression. A recent study in mammalian cells proposed that reduction of eIF2α phosphorylation is an adaptive mechanism that protects the cell from severe inhibition of protein synthesis under long-term mitochondrial stress (Samluk et al. 2019). This adaptive mechanism may also apply to the plant, since the plant with iMUCH condition suffers a long-term mild mitochondrial stress that is different from AA-induced short-term acute mitochondrial stress (Fig. 8). An adaptive response to a short-term acute mitochondrial stress via the TOR pathway allows the cell to sense cellular energy status to restore proteostasis to avoid cellular death (Fig. 8) (Wang & Chen 2015; Topf et al. 2018; Samluk et al. 2019). Whereas, under a long-term mitochondrial stress, the cell may benefit from eIF2α mediated feedback control of protein synthesis for survival, while the translation is selectively repressed due to the reduction of the components in the translation machinery (Fig. 6A, B). We assume that eIF2α regulators and targets in plants, like ATF4 and PERK kinase in mammalian cells, are involved in mitochondrial-cytosolic protein quality control mechanisms for coping with the long-term mitochondrial stress (Harding et al. 2000; Khan et al. 2017; Kuhl et al. 2017; Quiros et al. 2017; Fessler et al. 2020; Guo et al. 2020). Moreover, the selective translation has been considered for aiding recovery from mitochondrial stress (Nandagopal & Roux 2015; Pakos-Zebrucka et al. 2016; Shi et al. 2017; Gerst 2018; Topf et al. 2019). Therefore, identifying eIF2α regulators and targets and putative mechanisms for the selective repression of mRNA translation upon iMUCH will be an exciting area of future work.

We discover that iMUCH activates multi-compartmental UPR and cytosolic translation response, suggesting that iMUCH is likely a pathological condition that evokes the adaptive response as part of mitochondrial-cytosolic protein quality control mechanisms that have been unified as the mitoprotein-induced stress response (Boos et al. 2020). Although we are currently unable to unravel the full complexity of the iMUCH-evoked adaptive response at this stage, our findings in plants are merged with increasing evidence from studies in yeast and animals to support that mitochondrial stress-dependent regulation of protein synthesis has impacts on growth, stress resistance, and aging (Boos et al. 2020). iMUCH is linked to the inhibition of cell growth, which is a phenotype reminiscent of the inhibition of cell proliferation (Richter et al. 2013). The effects of iMUCH on growth are different from AA-mediated growth inhibition that is likely controlled by the TOR pathway in sensing cellular energy status (Fig. 8). Adaptive actions of the cell upon iMUCH may also include transcriptional and post-transcriptional co-suppression of cell growth pathways. On the other hand, iMUCH increases vulnerability to cellular senescence and stress. Cellular senescence is triggered by numerous stressors and physiological processes, including mitochondrial dysfunction, perturbed proteostasis, ribosomal stress, etc. (Gorgoulis et al. 2019). The hallmarks of senescence include deregulated metabolism, cell-cycle withdrawal, macromolecular damage, and secretory phenotypes. Whether these features mark 2OX and the sextuple mutant is an interesting question for future study. Nevertheless, iMUCH offers a pathological condition for studying how mitochondria regulate cellular senescence in vivo(Gorgoulis et al. 2019). While 2OX and the sextuple mutant show similar phenotypes in growth and stress resistance, the senescence occurs at a relatively late life stage in the sextuple mutant compared to 2OX. Given that the timing of senescence in 2OX plants is related to *MCU2* expression level, it is assumed that increasing mitochondrial Ca^2+^ uptake may accelerate cell-cycle arrest that is initiated by iMUCH. Therefore, 2OX plants show much earlier and more severe senescence than the sextuple mutant.

Finally, we acknowledge the limitations of this study despite our comprehensive characterization. First, while mitochondrial-targeted aequorin provides robust readouts of stimulus-induced _mt_Ca^2^⁺ dynamics, it lacks sufficient sensitivity to resolve basal (pre-stimulus) levels or subtle changes in _mt_Ca^2^⁺ and _cyt_Ca^2^⁺ following AA treatment. Consequently, we were unable to definitively compare basal Ca^2^⁺ homeostasis among wild type, 2OX, and sextuple mutant lines, should such differences exist. We recognize this constraint in our current study and cannot exclude the possibility of modest differences in basal _mt_Ca^2^⁺ homeostasis, which may be resolved using higher-sensitivity Ca^2^⁺ reporters in future investigations. Second, the upstream regulatory framework of MCU-mediated Ca^2^⁺ signaling remains undefined in this work. As mitochondrial Ca^2^⁺ uniporters primarily respond to elevated cytosolic Ca^2^⁺, MCU-dependent _mt_Ca^2^⁺ uptake functions downstream of osmotic stress- or salt stress-induced Ca^2^⁺ influx across the plasma membrane. Future genetic interaction analyses using established Ca^2^⁺ influx mutants (e.g., *osca* or *MOCA1* mutants) will help precisely position MCUs within the Ca^2^⁺ signaling hierarchy. Third, we focused our detailed analyses on a single, high-expression MCU2 overexpression line (2OX-1) to reliably capture molecular and physiological phenotypes linked to disrupted mitochondrial Ca^2^⁺ homeostasis. Although this strategy ensured clear and consistent phenotypic readouts, we cannot rule out functional divergence among individual MCU isoforms. Distinct MCUs may possess unique intrinsic activities or regulatory mechanisms, potentially leading to distinct functional outputs.

## Conclusion

In summary, our study identifies the mitochondrial Ca^2^⁺ uniporter as a central regulator of mitochondrial Ca^2^⁺ homeostasis in Arabidopsis and uncovers a novel cross-compartmental proteostatic response triggered by impaired MCU-controlled _mt_Ca^2^⁺ homeostasis. A key finding is that iMUCH activates a unique transcriptional program that upregulates nuclear genes linked to multi-compartment proteostasis. This response is distinct from the AA-induced mitochondrial retrograde response, as iMUCH does not elicit alternative oxidase-dependent respiration; instead, it engages a coordinated post-transcriptional program that represses the synthesis of ribosomal and RNA modification proteins to counterbalance transcriptional activation. We further demonstrate that iMUCH-induced translational repression is mediated by eIF2α phosphorylation, rather than the TOR pathway, representing an adaptive mechanism specific to long-term mild mitochondrial proteotoxic stress, as opposed to the acute stress triggered by AA. Notably, iMUCH impairs plant growth, accelerates senescence, and reduces osmotic stress tolerance, thereby establishing a direct link between _mt_Ca^2^⁺ homeostasis and stress resistance, a core focus of stress biology. Collectively, our results reveal an unreported connection between _mt_Ca^2^⁺ homeostasis and intercompartmental proteostasis, advancing our understanding of mitochondrial-nuclear communication under stress and providing potential genetic targets for improving crop resilience.

## Materials and methods

### Plant materials

All wild type, mutant, and transgenic Arabidopsis (*Arabidopsis thaliana*) lines used in this study were in the Columbia (Col-0) accession. Arabidopsis *MCU1* (At1g09575), *MCU2* (At1g57610), *MCU3* (AT2G23790), *MCU4* (At4g36820), *MCU5* (At5g42610), and *MCU6* (At5g66650) cDNAs were amplified by RT-PCR and cloned in-frame upstream of *YFP* into the binary vector pPZP200. About 1000 bp fragments encompassing the sequence upstream of the ATG were amplified from each *MCU* gene and cloned into the binary vector pMDC162. Single mutants *mcu1-1* (SALK_082151), *mcu2-1* (SALK_011710), *mcu3-1* (SALK_019312), *mcu4-1* (SALK_036975), and *mcu6-1* (SALK_037347) were obtained from the Arabidopsis Biological Resource Center (ABRC), and *mcu5-1* was generated using CRISPR/Cas9-mediated genome editing. The higher-order mutant *mcu1-1 mcu2-1 mcu3-1 mcu4-1 mcu5-1 mcu6-1* was generated using genetic crosses followed by identification of homozygous mutant plants from F_2_ or F_3_ progeny using PCR-based genotyping.

### Growth conditions for growth assessment

All Arabidopsis seedlings used to analyze growth phenotypes under normal or stress conditions were grown on half-strength Murashige-Skoog (MS, Caisson Labs) with 1% (w/v) sucrose medium solidified with 1.2% (w/v) agar supplemented with or without stressors. Briefly, Arabidopsis seeds were sown on MS plates. After stratification at 4 °C for 2 d in the dark, the plates were placed vertically in a growth chamber at 22 °C under 100 mmol m^−2^ s^−1^ fluorescent light (WT5-LED14, FSL) in a 16-h-light/8-h-dark photoperiod. Five-day-old seedlings were transferred to MS plates supplemented with or without 150 mM mannitol, and these plates were placed at 22 °C for 5–10 d before the phenotypes were recorded by taking photographs and measuring primary root length and fresh weight. In addition, 10-day-old seedlings were transferred to a 3″ square pot (4 plants per pot) or a 36-cell tray (1 plant per cell) filled with soil (75% commercial soilless germination mix and 25% calcined clay granules, 0.2–0.5 cm diameter) for the documentation of subsequent growth and development in a growth room with a temperature of 22ºC, a light intensity of 100–150 mmol m^−2^ s^−1^ fluorescent light (WT5-LED14, FSL) and 16-h-light/8-h-dark long-day photoperiodic conditions. Sub-irrigation was normally applied for young seedlings and for plants with maturing seeds. General-purpose liquid fertilizer w/micronutrients (150–200 mg N/liter) was supplied every other irrigation.

### Subcellular localization of MCU proteins and GUS staining

Subcellular localization of MCU proteins was examined using confocal microscopy (Zeiss 710) in the leaf and root cells of stable *35S:MCU-YFP* transgenic plants*.* YFP and chlorophyll fluorescence were excited at 514 nm with detection ranges of 525–600 nm and 650–720 nm for YFP and chlorophyll fluorescence, respectively. MitoTracker dye was excited at 644 nm with a detection range of 578–696 nm for MitoTracker™ Deep Red FM (Invitrogen M22426). For GUS staining, transgenic lines expressing *proMCUs:GUS* were used. Briefly, 10-day-old seedlings, rosette leaves, or flowers were harvested and placed in ice-cold 90% acetone for at least 2 h. Sample tissues were then placed under vacuum for 10 min at room temperature (RT) and left at RT for 20–30 min after the vacuum was released. Acetone was removed and replaced with GUS staining buffer (GSB: 100 mM sodium phosphate buffer, pH 7.0, 0.5 M EDTA, 10% Triton X-100, 50 mM potassium ferricyanide, and 50 mM potassium ferrocyanide) and placed under vacuum for 5 min. GSB was then replaced with GUS staining solution (GSS: GSB plus 2 mM X-GLUC). Sample tissues were incubated in GSS at 37 °C overnight. To visualize the blue precipitate in tissues, the GSS was removed, and tissues were immersed in 70% ethanol. Once cleared, tissues were mounted on a slide in a glycerol solution for observation or documented using a dissecting microscope.

### RNA isolation for cDNA amplification and RT-qPCR

Total RNA was extracted from less than 100 mg of 10-day-old seedlings that were grown vertically on MS plates, or treated with 50 µM AA or DMSO for 4 h in liquid MS. First-strand cDNA was synthesized using the HiScript® II cDNA Synthesis Kit (Vazyme R212-02), and oligo d(T) for the synthesis of cDNA used for amplification of nDNA encoded genes. qPCR with iTaq Universal SYBR® Green Supermix (BIORAD) was conducted on a Roche 480 Real-Time PCR system following the manufacturer’s instructions.

### Aequorin-based FAS imaging of _mt_Ca^2+^ and _cyt_Ca^2+^ response

For luminescence imaging of _mt_Ca^2+^ and _cyt_Ca^2+^, the two constructs *Ubpro:CytoAequorin-YFP* (*cytAeq,* CD3-1798) and *Ubpro:MitoAequorin-YFP* (*mtAeq,* CD3-1800) were used, consisting of the *Ubiquitin* promoter driving the expression of aequorin targeted to the cytosol and mitochondrial matrix, respectively (Mehlmer et al. 2012). These constructs were transformed into wild type Col-0, MCU2 overexpressing (2OX), and *mcu1-1 mcu2-1 mcu3-1 mcu4-1 mcu5-1 mcu6-1* sextuple mutant plants. Homozygous plants harboring cytAeq and mtAeq were subjected to salt and mannitol stimuli to record _mt_Ca^2+^ and _cyt_Ca^2+^ response using the aequorin-based FAS recording system (Zhu et al. 2013).Luminescence intensity of each FAS film containing about 30 seedlings was quantified by indiGO™ software, and converted to Ca^2+^ concentration by the formula pCa = 0.332588(-logk) + 5.5593, where k is a rate constant equal to stimulus-induced Ca^2+^ luminescence counts (CaL) divided by discharge luminescence counts (DisL), k = (CaL)/(DisL + CaL) (Knight et al. 1996; Zhu et al. 2013). For Ca^2+^ luminescence imaging by FAS assay, more than 6 independently prepared films for each genotype were recorded.

### Luminometry reading of _mt_Ca^2+^ and _cyt_Ca^2+^ response

For quantitative measurement of Ca^2+^ response using a luminometer (GLOMAX, Promega, USA, Model: 2031–002, Serial: 203,001,553), the 1.5 mL tube containing a single whole 8-day-old seedling incubated with coelenterazine solution (final concentration 5 μmol/L) overnight was set on the chamber for recording of the resting luminescence for about 60 s. Ca^2+^ luminescence value was read at 1 s intervals for another 100 s, starting by adding a final concentration of 100 mM NaCl or 400 mM mannitol into the tube. The remaining aequorin was discharged by adding the discharge buffer, and the luminescence value was read for 100 s. The _mt_Ca^2+^ dynamics of the wild type, 2OX, and sextuple mutant seedlings were performed using a luminometer. The luminescence value of each seedling was converted to Ca^2+^ concentration by the formula described above. For Ca^2+^ luminescence reading by a luminometer, 10–15 whole seedlings were independently measured. Ca^2+^ amplitudes, curve area, and peak time were calculated by comparing the mannitol or NaCl-induced _mt_[Ca^2+^]^peak^ against that of _mt_[Ca^2+^]^rest^. Average _mt_Ca^2+^ amplitude, curve area, and peak time were calculated from the data of independent measurements of 10–15 seedlings for each genotype. The area of _mt_Ca^2+^ response curve was determined by Graphpad Prism 9.

### RNA-seq and data analysis

Wild type (Col-0), 2OX, and sextuple mutant seedlings were grown vertically on half-strength MS plates. Ten-day-old whole seedlings were then transferred into 9-well plates containing half-strength liquid MS medium overnight with shaking in the dark, and then were treated with 50 µM AA or DMSO for 4 h. Total RNA was prepared using Trizol (Invitrogen). Sequencing libraries were generated from 1 µg RNA per sample using NEBNext® Ultra™ RNA Library Prep Kit for Illumina® (NEB, USA) following the manufacturer’s recommendations. The reads were then mapped against the *Arabidopsis thaliana* reference genome (TAIR10). Cufflinks v2.2.1 was used to obtain normalized reads per kilobase of transcript per million mapped reads (RPKM) values for each annotated gene. Three biological replicates of each treatment were used for RNA-seq analysis.

### GO enrichment analysis

The PANTHER website was used for GO term enrichment analysis of upregulated or downregulated genes (fold-change > 2, Benjamini–Hochberg adjusted *p*-value < 0.01) in RNA-seq data from Col-0, *2OX*, *4OX*, *6OX*, and the sextuple mutant treated with 50 µM AA. imageGP was used to generate GO enrichment plots and heatmaps.

### Immunoblotting

For preparation of total proteins, 10-day-old seedlings that were grown vertically on MS plates, or treated with 50 µM AA or DMSO in liquid MS for 4 h (~200 mg) were collected and ground to a fine powder in liquid nitrogen, then homogenized with two volumes of extraction buffer containing 125 mM Tris–HCl, pH 8.8, 1% (w/v) SDS, 10% (v/v) glycerol, and 50 mM Na_2_S_2_O_2_, 0.2 mM PMSF, and protease inhibitor cocktail (Roche). The supernatants were separated by 12.5% SDS-PAGE and immunoblotted with antibodies (Supplementary Table S6).

For preparation of enriched mitochondrial proteins, 10-day-old seedlings (~5 g) were ground in liquid nitrogen and extracted in 30 mL extraction buffer (15 mM MOPs, pH 7.4, 450 mM sucrose, 0.6% [w/v] PVP40, 0.2% [w/v] BSA, 1.5 mM EGTA, 10 mM DTT, 0.2 mM PMSF, and protease inhibitor cocktail). Homogenates were then filtered through two layers of Miracloth and centrifuged at 3,500 g for 10 min, followed by additional centrifugations at 8,000 g for 5 min and at 17,000 g for 30 min at 4ºC. The resulting pellets were washed with 1 mL washing buffer (0.3 M sucrose, 15 mM MOPS, pH 7.2) twice. Pellets were resuspended in 200 µL extraction buffer for immunoblotting with antibodies (Supplementary Table S6).

### Proteomic analysis

Ten-day-old whole seedlings of wild type (Col-0) and sextuple mutant seedlings grown vertically on half-strength MS plates were collected. For AA treatment, ten-day-old whole seedlings of Col-0 were then transferred into 9-well plates containing half-strength liquid MS medium overnight with shaking in the dark, then treated with 50 µM AA or DMSO for 4 h. Whole seedlings (100 mg) were homogenized in lysis buffer consisting of 2.5% SDS/100 mM Tris–HCl (pH 8.0). TMT labeling was performed according to the manufacturer's instructions. LC–MS/MS data acquisition was carried out on an Orbitrap Exploris 480 mass spectrometer coupled with an Easy-nLC 1200 system. MS raw data were analyzed with MaxQuant (V1.6.6) using the Andromeda database search algorithm. Spectra files were searched against the UniProt *Arabidopsis thaliana* proteome database. Search results were filtered with 1% FDR at both protein and peptide levels.

### Polysome profiling and TE calculations

Sample preparation, collection and AA treatment were performed as described in proteomic analysis. Whole seedlings (~200 mg) were ground in liquid nitrogen and extracted in 1 mL polysome buffer (400 mM Tris–HCl, pH 8.4, 100 mM MgCl_2_, 200 mM KCl, 0.5% [v/v] NP-40, 50 µM EGTA, 50 µg/mL cycloheximide, 50 µg/mL chloramphenicol, and 400 U/mL recombinant RNasin [RNase inhibitor, Promega]). The supernatant (1 mL) was loaded on top of the sucrose gradient. The gradients were ultracentrifuged for 3 h at 175,000 g in an SW41Ti rotor (Beckman-Coulter, USA) and analyzed by measuring continuous absorbance at 260 nm using a Piston Gradient Fractionator (Biocomp Instruments). Total RNA of polysome or monosome fractions was precipitated for RNA-seq analysis, and the data analysis was performed as described previously (Mustroph et al. 2009; Missra & von Arnim 2014).

TEs were calculated as the ratio between the transcripts in the polysome fractions (highly translated ribosome-mRNA fractions) and the transcripts in the monosome fractions (lower translated ribosome-mRNA fractions) (Molenaars et al. 2020). TEs were then compared between *2OX* or sextuple mutant samples and wild type control samples as defined: log_2_ [(MCU^polysome^/MCU^monosome^)/(WT^polysome^/WT^monosome^)]. Monosome and polysome fractions were collected as indicated in Fig. 5. The means of three replicates were used to calculate the ratios.

## Supplementary Information

Supplementary Material 1: Fig. S1 Generation of *mcu* mutants and Ca^2+^ reporter lines. A. Schematic diagrams show the positions of the T-DNA insertions within *MCU1*, *MCU2*, *MCU3*, *MCU4*, and *MCU6* and the CRISPR/Cas9-edited sites within *MCU5.*B. The DNA gel image shows PCR confirmation of mutant lines. G stands for gene-specific primer pairs for detecting the presence of a gene, and T stands for T-DNA-specific primer pairs for detecting the presence of T-DNA. C. Relative transcript levels of *MCU*s in the sextuple mutant. MCU transcripts were detected in the sextuple mutant using RT-qPCR. The numbers above the columns indicate log_2_ fold-changes in transcripts of sextuple mutants relative to the wild type. D. Relative transcript levels of *MCU*s in Col*:mtAq*, *2OX:mtAq* and *mcu*^*1-6*^*:mtAq* lines. MCU transcripts were detected by RT-qPCR. The numbers above the columns indicate log_2_ fold-changes in transcripts of mutants or overexpression lines relative to the wild type. Results are expressed as the mean ± SEM (*n* = 3). A two-tailed Student’s *t*-test was performed, *P < 0.05, *P < 0.01, ***P < 0.001, and ****P < 0.0001. See also Supplementary Table S7 for the information on statistical analysis. Fig. S2 MCU transcript levels in *MCU* overexpression lines. Relative transcript levels of *MCU2*, *MCU4*, and *MCU6* in *2OX*, *4OX*, and *6OX* lines, determined from the relative transcript levels of *YFP* by RT-qPCR. Results are expressed as the mean ± SEM (*n* = 3). A two-tailed Student’s *t*-test was performed to examine statistical significance, ****P < 0.0001. See also Supplementary Table S7 for the information on statistical analysis. Fig. S3 Translational repression of *cyRP*s. A and B. Levels of individual *cyRP* transcripts and TEs are plotted, showing a large number of upregulated *cyRP* transcripts with low TE. The percentage of upregulated *cyRP* transcripts (A) and *cyPR* mRNAs with low TE (B) is indicated in each plot. See also the detailed information in Supplementary Table S5. Fig. S4 GO enrichment of the genes with saTE in AA-treated wild type. Top six biological processes with highest enrichment scores in low TE (blue) or high TE (yellow) transcripts for each GO category. See also Supplementary Table S5 for the information on GO terms. Fig. S5 Abundance of OXPHOS subunits is altered in both gain-of-function and knockdown-of-function *MCU* plants. A-D. Comparison of OXPHOS subunit protein abundance. nDNA subunits (labeled as n) and mtDNA subunits (labeled as mt) of OXPHOS complexes I (A), III (B), IV (C), and V (D) were detected and quantified by immunoblotting using antibodies against each subunit of complex l: NAD2, NAD5, and CA3; lll: COB and CYC1-1; IV: COX1, COX3, and COX X1; and V: ATP4, ATP8, and ATP2. Quantifications of signals relative to the wild type (set to 100%) are provided below each panel as means ± SEM (*n* = 3). A two-tailed Student’s *t*-test was performed to examine statistical significance, *p < 0.05, **p< 0.01, with the fold change (vs wild type) |FC| > 1.2. See also Supplementary Table S6 for the information on antibodies and primers and Supplementary Table S7 for the information on statistical analysis. Fig. S6 GO enrichment of the DEGs in 2OX and the sextuple mutant. A and B. Top biological processes, molecular function, and cellular component in upregulated DEGs (A) or downregulated DEGs (B) are listed for each GO category. The filtering criteria for DAPs are |FC| > 1.5 and padj < 0.05. See also Supplementary Table S1 for the information on GO terms.

Supplementary Material 2: Table S1. Differentially expressed genes identified in 2OX and sextuple mutant and AA-treated wild type.

Supplementary Material 3: Table S2. Expression of genes important for mitochondrial proteostasis in 2OX and the sextuple mutant.

Supplementary Material 4: Table S3. Mass spectrometry proteomics data.

Supplementary Material 5: Table S4. The list of genes for proteome–transcriptome cross-analysis.

Supplementary Material 6: Table S5. The monosomal and polysomal transcripts for analysis of translation efficiency.

Supplementary Material 7: Table S6. Primer sets and antibodies.

Supplementary Material 8: Table S7. Statistical information.

## Data Availability

The data supporting our findings can be found in the supplementary materials. The RNA-seq and Ribo-seq data generated in this study have been deposited in Gene Expression Omnibus (GEO) under the accession number GSE186418. The mass spectrometry proteomics data have been deposited to the ProteomeXchange Consortium (http://proteomecentral.proteomexchange.org) via the iProX partner repository (Ma et al. 2019) with the Table identifier PXD029171.

## Abbreviations

AA: Antimycin A
AOX: Alternative Oxidase
ABCF4: ABC transporter F family member 4
ABCF5: ABC transporter F family member 5
cpHsp: Chloroplast Chaperones
cpRP: Chloroplast Ribosome Protein
cyHsp: Cytosolic Chaperones
cyRP: Cytosolic Ribosome Protein
DAP: Differentially Abundant Proteins
EMRE: Essential MCU Regulator
eIF2α: Eukaryotic Initiation Factor 2α
eIF2-A2: Eukaryotic Initiation Factor 2α subunit
eIF3d: Eukaryotic Initiation Factor 3d
EIF3: Eukaryotic Initiation Factor 3
ETC: Electron Transport Chain
erHsp: Endoplasmic Reticulum Chaperones
FAS: Film Adhesive Seedling
X-GLUC: 5-Bromo-4-chloro-3-indolyl-β-D-glucuronide dissolved in N,N-dimethylformamide
GUS: β-Glucuronidase
KIN10: SNF1 kinase homolog 10
p-KIN10: Phosphorylated Form of KIN10
KIN11: SNF1 kinase homolog 11
p-KIN11: Phosphorylated Form of KIN11
iMUCH: Impaired MCU-controlled mtCa^2^⁺ Homeostasis
JA: Jasmonic Acid
MCU: Mitochondrial Ca^2^⁺ Uniporter
MCUC: Mitochondrial Ca^2^⁺ Uniporter Protein Complex
MICU: Subunit of MCUC
MS: Murashige-Skoog
Msn2/4: Msn2/4 transcription factor
mTOR: Mechanistic Target of Rapamycin
mtCa^2^⁺: Mitochondrial matrix Ca^2^⁺
mtHsp: Mitochondrial Chaperones
mtRP: Mitochondrial Ribosome Protein
mtDNA: Mitochondrial DNA
COB: MtDNA-encoded Cytochrome b Protein
COX1: MtDNA-encoded Cytochrome c Oxidase Subunit 1 Protein
ATP4: MtDNA-encoded ATP Synthase Subunit 4 Protein
ATP8: MtDNA-encoded ATP Synthase Subunit 8 Protein
NAD2: Mitochondrial Complex I Subunit NAD2 (NADH Dehydrogenase Subunit 2)
NAD5: Mitochondrial Complex I Subunit NAD5 (NADH Dehydrogenase Subunit 5)
nDNA: Nuclear DNA
CA3: NDNA-encoded Carbonic Anhydrase 3 Protein
COX X1: NDNA-encoded Cytochrome c Oxidase Subunit X1 Protein
CYC1: NDNA-encoded Cytochrome c1 Protein
ATP2: NDNA-encoded ATP Synthase Subunit 2 Protein
MRR: Mitochondrial Retrograde Response
PCD: Programmed Cell Death
PLS-DA: Partial Least-Squares Discriminant Analysis
PPR: Pentatricopeptide Repeat Proteins
PUS: Pseudouridine Synthases
RP: Ribosome Protein
saTE: Significantly Altered TE
TE: Translational Efficiency
TOR: Target of Rapamycin
TPR: Tetratricopeptide Repeat Proteins
TRM61: TRM61 Protein (tRNA Methyltransferase 61)
NagB: NagB Protein (N-Acetylglucosamine-6-Phosphate Deaminase B)
ROS: Reactive Oxygen Species
UPR: Unfolded Protein Response
UPRcyt: Cytosolic Unfolded Protein Response
UPRchl: Chloroplast Unfolded Protein Response
UPRer: Endoplasmic Reticulum Unfolded Protein Response
UPRmt: Mitochondrial Unfolded Protein Response
UPRs: Unfolded Protein Responses
VIP: Variable Importance in Projection
