# Supplementary material for: Perturbation of mitochondrial Ca2+ homeostasis activates cross-compartmental proteostatic response in Arabidopsis
Source: Stress Biol. 2026 May 28;6(1):40. doi: 10.1007/s44154-026-00314-4 (PMC13219543; doi:10.1007/s44154-026-00314-4)
Supplement: Supplementary file 1 — Supplementary Material 1: Fig. S1 Generation of mcu mutants and Ca2+ reporter lines. A. Schematic diagrams show the positions of the T-DNA insertions within MCU1, MCU2, MCU3, MCU4, and MCU6 and the CRISPR/Cas9-edited sites within MCU5.B. The DNA gel image shows PCR confirmation of mutant lines. G stands for gene-specific primer pairs for detecting the presence of a gene, and T stands for T-DNA-specific primer pairs for detecting the presence of T-DNA. C. Relative transcript levels of MCUs in the sextuple mutant. MCU transcripts were detected in the sextuple mutant using RT-qPCR. The numbers above the columns indicate log2 fold-changes in transcripts of sextuple mutants relative to the wild type. D. Relative transcript levels of MCUs in Col:mtAq, 2OX:mtAq and mcu1-6:mtAq lines. MCU transcripts were detected by RT-qPCR. The numbers above the columns indicate log2 fold-changes in transcripts of mutants or overexpression lines relative to the wild type. Results are expressed as the mean ± SEM (n = 3). A two-tailed Student’s t-test was performed, *P < 0.05, *P < 0.01, ***P < 0.001, and ****P < 0.0001. See also Supplementary Table S7 for the information on statistical analysis. Fig. S2 MCU transcript levels in MCU overexpression lines. Relative transcript levels of MCU2, MCU4, and MCU6 in 2OX, 4OX, and 6OX lines, determined from the relative transcript levels of YFP by RT-qPCR. Results are expressed as the mean ± SEM (n = 3). A two-tailed Student’s t-test was performed to examine statistical significance, ****P < 0.0001. See also Supplementary Table S7 for the information on statistical analysis. Fig. S3 Translational repression of cyRPs. A and B. Levels of individual cyRP transcripts and TEs are plotted, showing a large number of upregulated cyRP transcripts with low TE. The percentage of upregulated cyRP transcripts (A) and cyPR mRNAs with low TE (B) is indicated in each plot. See also the detailed information in Supplementary Table S5. Fig. S4 GO enrichment of the gene [file 44154_2026_314_MOESM1_ESM.pdf]

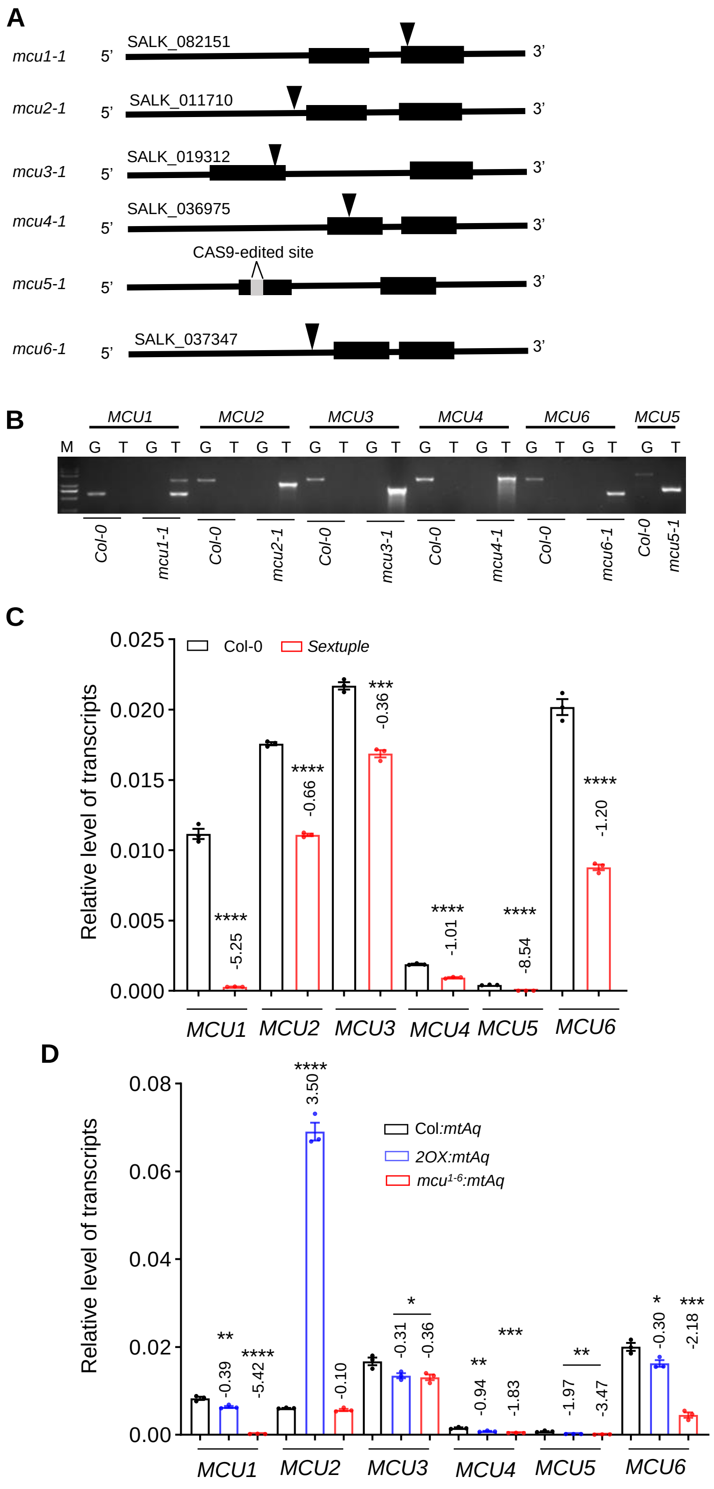

Fig. S1 Generation of *mcu* mutants and Ca^{2+} reporter lines. A. Schematic diagrams show the positions of the T-DNA insertions within *MCU1*, *MCU2*, *MCU3*, *MCU4*, and *MCU6* and the CRISPR/Cas9-edited sites within *MCU5.* B. The DNA gel image shows PCR confirmation of mutant lines. G stands for gene-specific primer pairs for detecting the presence of gene; and T stands for T-DNA specific primer pairs for detecting the presence of T-DNA. C. Relative transcript levels of *MCU*s in sextuple mutant. MCU transcripts were detected in sextuple mutant using RT-qPCR. The numbers above the columns indicate log2 fold-changes in transcripts of sextuple mutants relative to the wild type. D. Relative transcript levels of *MCU*s in Col*:mtAq*, *2OX:mtAq* and *mcu*$^{1\text{-}6}$*:mtAq* lines. MCU transcripts were detected by RT-qPCR. The numbers above the columns indicate log2 fold-changes in transcripts of mutants or overexpression lines relative to the wild type. Results are expressed as the mean ± SEM (n = 3). A two-tailed Student's *t*-test was performed, *P < 0.05, **P < 0.01, ***P < 0.001, and ****P < 0.0001. See also Supplementary Table S7 for the information on statistical analysis.

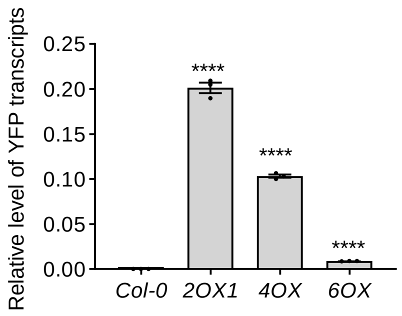

Fig. S2 MCU transcript levels in *MCU* overexpression lines. Relative transcript levels of *MCU2*, *MCU4*, and *MCU6* in *2OX*, *4OX*, and *6OX* lines, determined from the relative transcript levels of *YFP* by RT-qPCR. Results are expressed as the mean ± SEM (n = 3). A two-tailed Student's *t*-test was performed to examine statistical significance, ****$P < 0.0001$. See also Supplementary Table S7 for the information on statistical analysis.

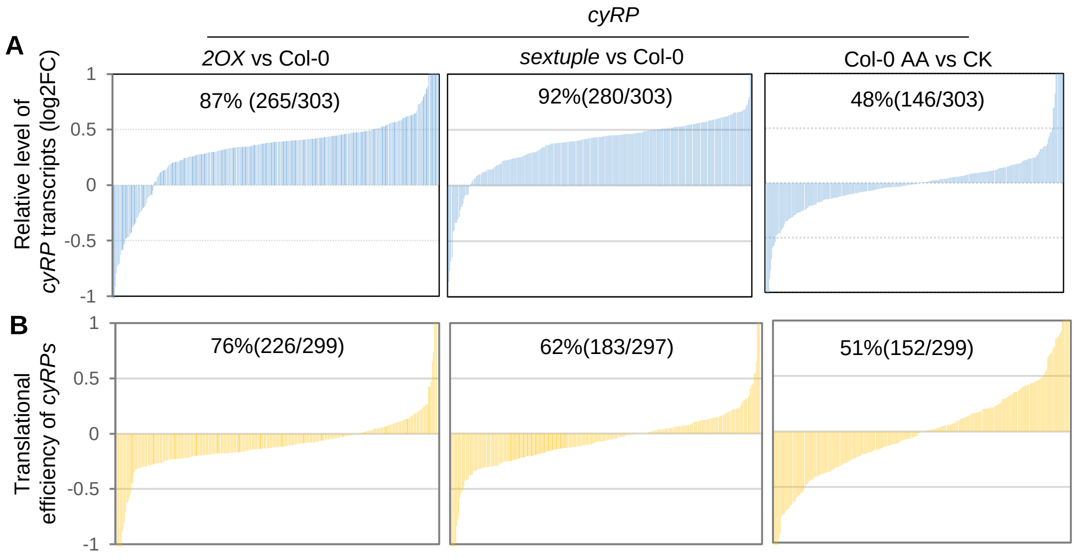

Fig. S3 Translational repression of *cyRP*s. A and B. Levels of individual *cyRP* transcripts and TEs are plotted, showing a large number of upregulated *cyRP* transcripts with low TE. The percentage of upregulated *cyRP* transcripts (A) and *cyPR* mRNAs with low TE (B) are indicated in each plot. See also the detailed information in Supplementary Table S5.

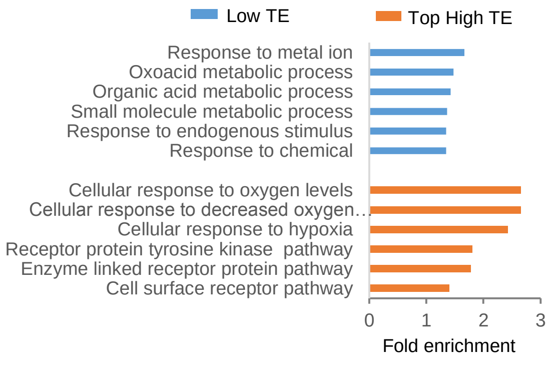

Fig. S4 GO enrichment of the genes with saTE in AA-treated wildtype. Top six biological processes with highest enrichment scores in low TE (blue) or high TE (yellow) transcripts for each GO category. See also Supplementary Table S5 for the information on GO terms

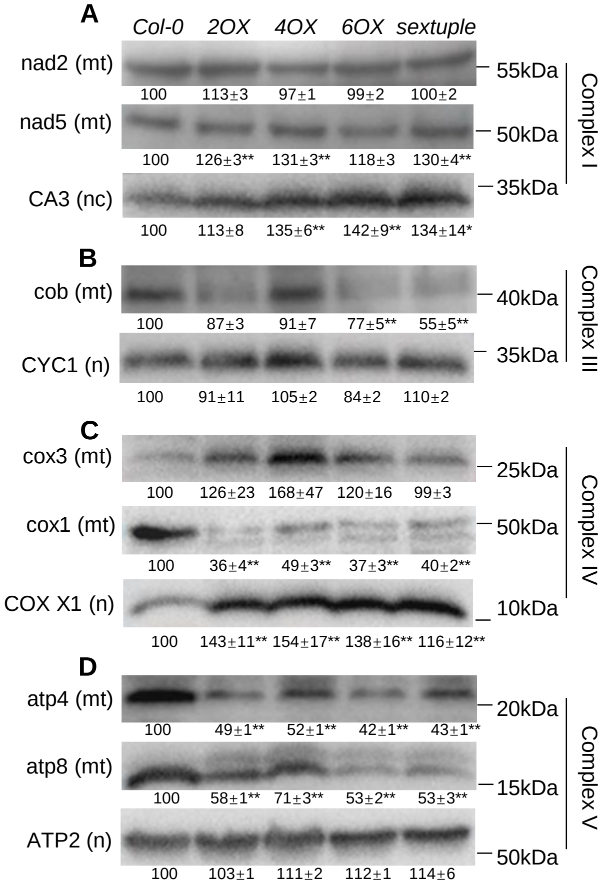

Fig. S5 Abundance of OXPHOS subunits is altered in both gain-of-function and loss-of-function *MCU* plants. A-D. Comparison of OXPHOS subunit protein abundance. nDNA subunits (labeled as n) and mtDNA subunits (labeled as mt) of OXPHOS complexes I (A), III (B), IV (C), and V (D) were detected and quantified by immunoblotting using antibodies against each subunit of complex I: NAD2, NAD5 and CA3, III: COB and CYC1-1, IV: COX1, COX3 and COX X1, and V: ATP4, ATP8 and ATP2. Quantifications of signals relative to the wildtype (set to 100%) are provided below each panel as means ± SEM (n = 3). A two-tailed Student's t-test was performed to examine statistical significance, *p < 0.05, **p < 0.01 with the fold change (vs wildtype) |FC| > 1.2. See also Supplementary Table S6 for the information on antibodies and primers, and Supplementary Table S7 for the information on statistical analysis.

A

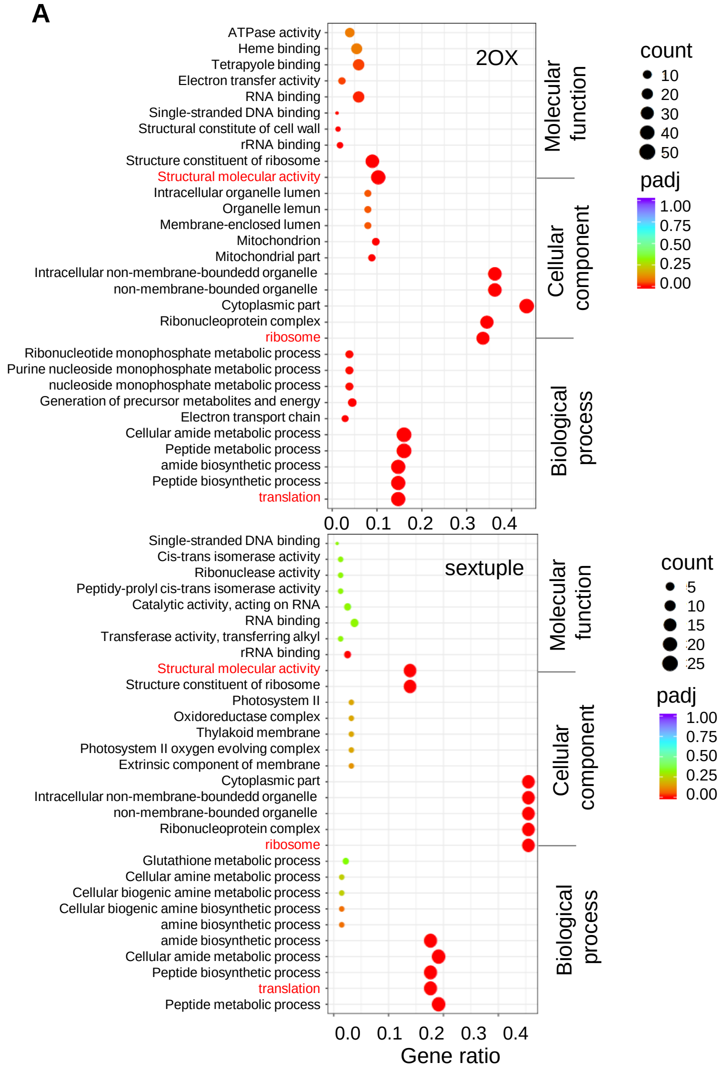

2OX
ATPase activity
Heme binding
Tetrapyole binding
Electron transfer activity
RNA binding
Single-stranded DNA binding
Structural constitute of cell wall
rRNA binding
Structure constituent of ribosome
Structural molecular activity
Intracellular organelle lumen
Organelle lemun
Membrane-enclosed lumen
Mitochondrion
Mitochondrial part
Intracellular non-membrane-boundedd organelle
non-membrane-bounded organelle
Cytoplasmic part
Ribonucleoprotein complex
ribosome
Ribonucleotide monophosphate metabolic process
Purine nucleoside monophosphate metabolic process
nucleoside monophosphate metabolic process
Generation of precursor metabolites and energy
Electron transport chain
Cellular amide metabolic process
Peptide metabolic process
amide biosynthetic process
Peptide biosynthetic process
translation
0.0 0.1 0.2 0.3 0.4
Molecular function
Cellular component
Biological process
count
10
20
30
40
50
padj
1.00
0.75
0.50
0.25
0.00
sextuple
Single-stranded DNA binding
Cis-trans isomerase activity
Ribonuclease activity
Peptidy-prolyl cis-trans isomerase activity
Catalytic activity, acting on RNA
RNA binding
Transferase activity, transferring alkyl
rRNA binding
Structural molecular activity
Structure constituent of ribosome
Photosystem II
Oxidoreductase complex
Thylakoid membrane
Photosystem II oxygen evolving complex
Extrinsic component of membrane
Cytoplasmic part
Intracellular non-membrane-boundedd organelle
non-membrane-bounded organelle
Ribonucleoprotein complex
ribosome
Glutathione metabolic process
Cellular amine metabolic process
Cellular biogenic amine metabolic process
Cellular biogenic amine biosynthetic process
amine biosynthetic process
amide biosynthetic process
Cellular amide metabolic process
Peptide biosynthetic process
translation
Peptide metabolic process
0.0 0.1 0.2 0.3 0.4
Gene ratio
Molecular function
Cellular component
Biological process
count
5
10
15
20
25
padj
1.00
0.75
0.50
0.25
0.00

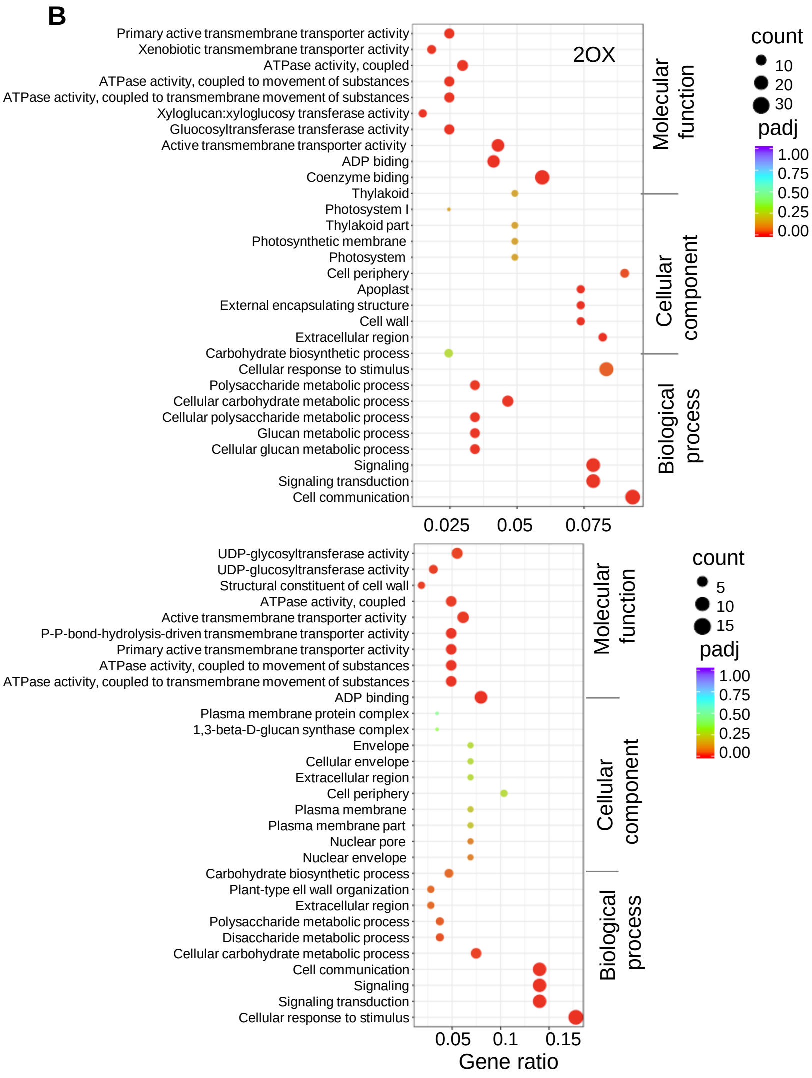

Fig. S6 GO enrichment of the DEGs in 2OX and sextuple mutant. (a) and (b)Top biological process, molecular function and cellular component in upregulated DEGs (a) or downregulated DEGs (b) are listed for each GO category. The filtering criteria for DAPs are |FC| > 1.5 and padj < 0.05. See also Dataset S1 for the information on GO terms.
